# Terrestrial evidence for ocean forcing of Heinrich events and subglacial hydrologic connectivity of the Laurentide Ice Sheet

**DOI:** 10.1126/sciadv.abp9329

**Published:** 2022-10-19

**Authors:** Graham H. Edwards, Terrence Blackburn, Gavin Piccione, Slawek Tulaczyk, Gifford H. Miller, Cosmo Sikes

**Affiliations:** ^1^ Department of Earth Sciences, Dartmouth College, Hanover, NH 03755, USA.; ^2^ Department of Earth and Planetary Sciences, University of California Santa Cruz, Santa Cruz, CA 95064, USA.; ^3^ Institute of Arctic and Alpine Research and the Department of Geological Sciences, University of Colorado, Boulder, CO 80309, USA.; ^4^ Department of Geology, University of Maryland, College Park, MD 20742, USA.

## Abstract

During the last glacial period, the Laurentide Ice Sheet (LIS) underwent episodes of rapid iceberg discharge, recorded in ocean sediments as “Heinrich events” (HEs). Two competing models attempt to describe the stimulus for HEs via either internal ice sheet oscillations or external ocean-climate system forcing. We present a terrestrial record of HEs from the northeastern LIS that strongly supports ocean-climate forcing. Subglacial carbonate precipitates from Baffin Island record episodes of subglacial melting coincident with the three most recent HEs, resulting from acceleration of nearby marine-terminating ice streams. Synchronized ice stream acceleration over Baffin Island and Hudson Strait is inconsistent with internal ice sheet oscillations alone and indicates a shared ocean-climate stimulus to coordinate these different glaciological systems. Isotopic compositions of these precipitates record widespread subglacial groundwater connectivity beneath the LIS. Extensive basal melting and flushing of these aquifers during the last HE may have been a harbinger for terminal deglaciation.

## Teaser

Subglacial mineral precipitates record ocean forcing of Heinrich events and widespread subglacial groundwater connectivity.

## INTRODUCTION

Over the course of the last 120 thousand years (ka), continental ice volumes and glacial climate conditions peaked during the last glacial maximum (LGM; ca. 26.5 to 19 ka ago) (*1*, *2*), followed by collapse of the Eurasian ice sheets and North American Laurentide Ice Sheet (LIS) and transition to an interglacial climate. Superimposed on these orbitally paced climate trends were millennial-scale oscillations in climate and ice dynamics linked to interactions among the ice-ocean-atmosphere systems. For instance, the so-called Dansgaard-Oeschger (D-O) events, originally observed in the oxygen isotope records of Greenland ice cores, reflect episodes of pronounced local atmospheric warming (*3*). D-O warm phases, or interstadials, are characterized by warm Greenland air temperatures, limited continental ice and freshwater flux into the North Atlantic, and strong North Atlantic deepwater (NADW) formation and Atlantic meridional overturning circulation (AMOC). D-O cold phases, or stadials, are instead characterized by cooler Greenland air temperatures, enhanced continental ice and freshwater flux to the North Atlantic, and dampened NADW formation and AMOC. Superimposed over some stadials, but not all, are Heinrich events (HEs): pulses of widespread iceberg discharge into the North Atlantic, predominantly from the LIS, that coincide with particularly fresh North Atlantic surface waters and especially weak NADW and AMOC (*3*, *4*). HEs are apparently linked to stadial conditions and occurred only during stadials and correlated climate proxies (*5*).

**Fig. 1.**
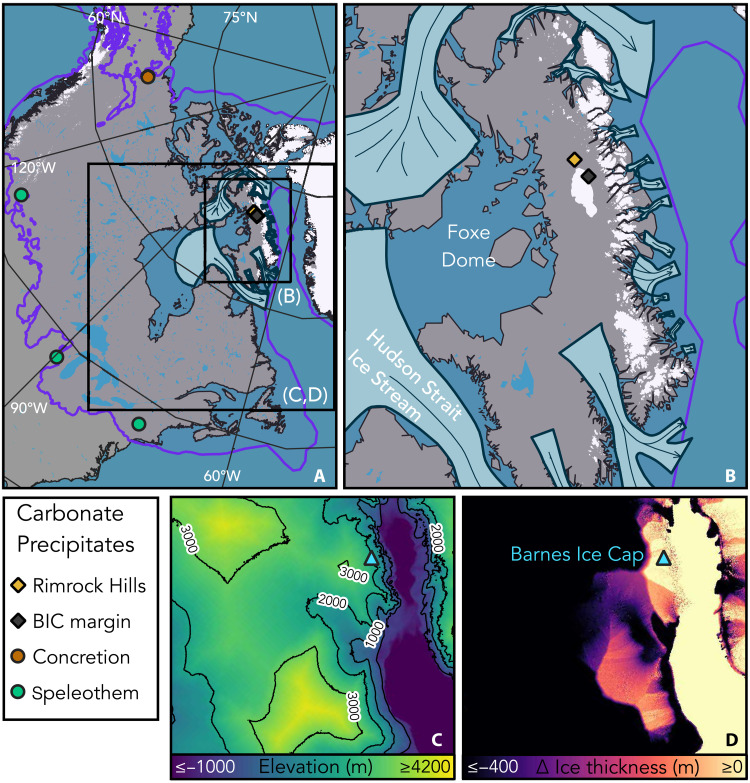
Modern and LGM conditions of northern North America and Baffin Island. (**A** and **B**) Modern sea level and land-ice distribution (white) are overlain by LGM Laurentide Ice Sheet extent (purple border) (*73*), paleo-ice streams draining the Foxe Dome (pale blue with dark borders) (*33*), subglacial carbonate precipitates measured in this study (diamonds), and groundwater-fed periglacial carbonate precipitates with δ^234^U_o_ >500‰ (circles) (*47*, *58*–*60*). Black boxes in (A) indicate extents of (B) to (D). (**C**) Reconstructed ice surface elevation and surrounding land and bathymetry (relative to modern sea level) at 20 ka ago (*35*). (**D**) Change in reconstructed ice thickness between 20 and 12.5 ka ago (*35*).

HEs were first identified as intervals of increased lithic grain abundances in deep-sea sediment cores from the North Atlantic that reflect deposition of ice-rafted detritus (IRD) dispersed widely by icebergs (*6*, *7*). They recur quasiperiodically every ca. 7 ka, and provenance studies have traced the bulk of HE-associated IRD to the Hudson Strait, implicating the Hudson Strait Ice Stream (HSIS; Fig. 1) as the predominant source of HE iceberg discharge, although other IRD sources do occur and are more prominent in some HE layers (*4*). Despite the unmistakable presence of HEs in ocean sedimentary archives and correlated climate proxies, the mechanisms initiating HSIS surging and iceberg swarms in the North Atlantic are still under debate. The quasiperiodicity and heterogeneous behavior of HEs (*4*) have motivated a longstanding effort to unravel their spatiotemporal complexity with a coherent mechanistic model. The onset of HEs depend on some combination of “internal” ice sheet dynamics and “external” ocean-climate forcings that enhanced ice streaming and iceberg production by the HSIS.

MacAyeal (*8*) proposed a model to describe HE cyclicity that is regulated entirely by internal ice sheet thermodynamics. Under this model, the HSIS begins in a state of reduced thickness and frozen basal conditions, resulting in slow ice discharge that causes the LIS to thicken. Eventually, ice thickness becomes sufficient to drive basal melting, which lubricates soft deformable sediments at the bed, causing surging of the HSIS and concomitant iceberg discharge to the North Atlantic, until the LIS thins to the initial condition. While this internal dynamics model emphasizes the importance of HSIS basal conditions and ice streaming in facilitating rapid release of icebergs into the North Atlantic, the external ocean-climate forcing model underscores the temporal coherence of HEs with fluctuations in the ocean-climate system. The observation that AMOC weakening not only overlaps HE-IRD layers but both precedes and follows them suggests that HE iceberg surging is a response to an ocean-climate forcing, rather than an internally paced ice sheet process (*9*). Marcott *et al.* (*10*) showed that reduced AMOC circulation, as during a stadial, led to convective mixing of low-latitude warm waters that propagated into the North Atlantic subsurface, where intermediate-depth warming maxima coincided with HEs. In their model (*10*), these warm waters came into contact with and melted ice shelves, which undermined their capacity to buttress ice streams and resulted in ice stream surging and rapid ice loss. Given the evidence for persistent sea-ice and open-water conditions in Baffin Bay (*11*) and the Labrador Sea (*12*) rather than extended ice shelf cover, Bassis *et al.* (*13*) emphasized the vulnerability of ice stream grounding lines to ocean warming. By modeling HSIS surging in response to grounding line melting modulated by glacial isostatic adjustment, they reproduced the differential tempos of HEs relative to D-O oscillations (*13*). These ocean-climate forced models are corroborated by recent work showing that North Atlantic IRD deposition began during peak subsurface warming (*14*).

HEs entailed the loss of large volumes of continental ice as meltwater and icebergs (*4*, *15*, *16*). Of particular importance is the final HE (H1, with progressively earlier events increasing in number), which coincided with the beginning of the last glacial termination. H1 entailed multiple phases of complex ice-ocean interactions spanning as much as 4 ka (*17*) and may have actively exacerbated terminal ice sheet collapse (*18*). During the time frame of H1 iceberg discharge and IRD deposition, the δ^234^U (calculated as δ^234^U = 1000×[(^234^U/^238^U) − 1], where parentheses denote the activity ratio) of Atlantic surface waters rose by several per mille, indicating the influx of high-δ^234^U terrestrial waters, likely sourced from subglacial reservoirs (*19*). The precise subglacial source of this high-δ^234^U deglacial run-off is unclear, but its probable subglacial provenance temporally correlates ice sheet processes with H1 iceberg surging and terminal LIS collapse. A better understanding of the subglacial source of these distinct waters would clarify if these events are related and, if so, what glaciological mechanisms connected them. Like the North Atlantic IRD layers that characteristically record HEs, the Atlantic δ^234^U record, in this case from deep-sea corals, is a marine record linked to LIS perturbations during episodes of climate change. Connecting these marine archives to specific glaciological systems and processes is crucial for accurately reconstructing the glacial and deglacial histories of ice sheets.

To date, our current understanding of LIS dynamics over the course of HE and D-O cycles come from mathematical simulations, ocean sediment cores, and distal climate proxy records (*5*, *13*, *20*). Subglacial erosion and postglacial flooding of the HSIS and other ice stream beds (*21*) have overprinted or obscured in situ terrestrial evidences of LIS instability during HEs. On Baffin Island, however, the arid polar climate and persistence of cold-based ice caps have preserved subglacial calcite mineral precipitates near the margin of the modern-day Barnes Ice Cap (BIC) and the Rimrock Hills region (Fig. 1). Prior studies of these precipitates verified their subglacial origin and dated their formation approximately contemporaneous with the LGM (*22*, *23*). These aqueous precipitates require the presence of liquid water supersaturated with respect to calcite at a location that has previously been interpreted to be beneath cold-based ice (*24*). Acceleration of marine-terminating ice streams that drained the Foxe Dome of the LIS into Baffin Bay (Fig. 1) may have produced sufficient basal shear heating (*25*, *26*) to drive the requisite subglacial melting. Here, we report U-Th dates of multiple episodes of aqueous carbonate precipitation on Baffin Island and assess the provenance of the calcite-forming waters with stable and radiogenic isotope proxies. We propose that these subglacial precipitates record changes in the basal thermal regime due to HE-related ice streaming, supported by both the time scales of carbonate precipitation and the subglacial hydrologic systems in which they formed. By contextualizing these carbonate-forming waters relative to potential subglacial aquifers of the LIS, we substantiate a model of an expansive and broadly connected LIS subglacial groundwater system that was evacuated into marine basins by extensive basal melting during the early stages of deglaciation and H1.

## RESULTS

### Synchrony of HEs and subglacial calcite formation on Baffin Island

**Fig. 2.**
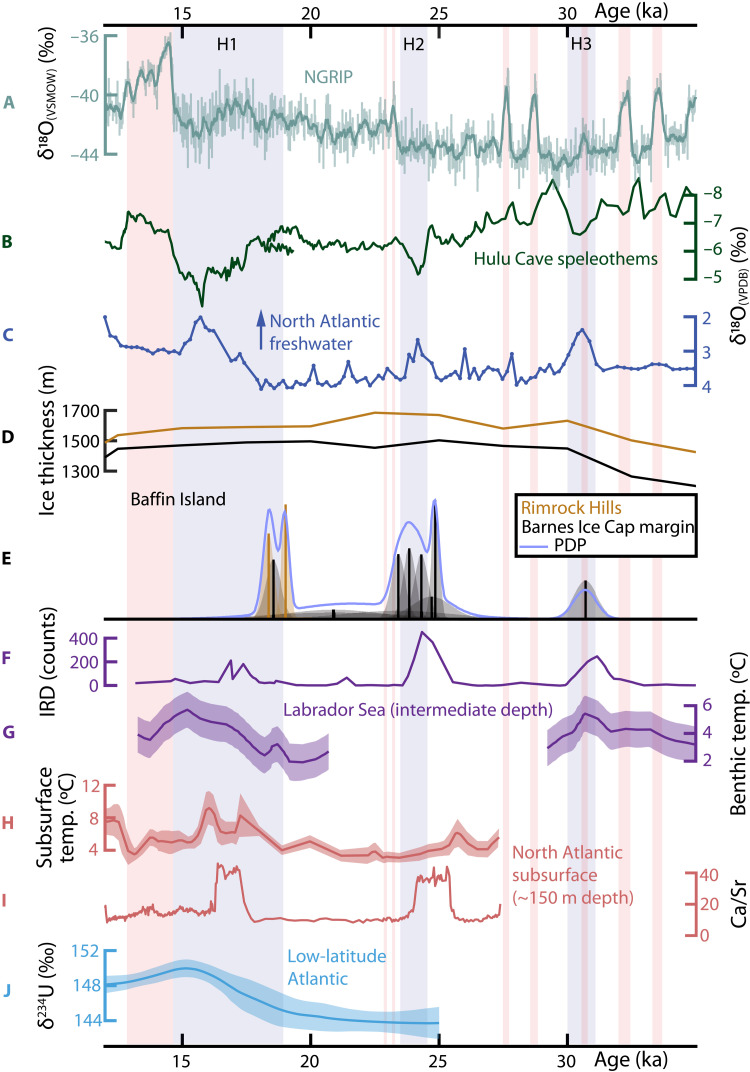
Chronologic comparison of Baffin Island subglacial calcite-forming events with various climate, ocean, and ice sheet records. Datum: 1950 CE. Proxies from the same record share the same color. Pale red bars indicate “Greenland Interstadial” events (*75*). Pale blue bars indicate HEs: H1 spans the comprehensive time frame of (*17*) and H2 and H3 span associated excursions in the Hulu Cave speleothem record (B). (**A**) NGRIP δ^18^O record of Greenland temperature variation (GICC05 time scale, adjusted to 1950 CE datum) (*75*). (**B**) δ^18^O of Hulu Cave stalagmites (PD and MSD) record Asian monsoon precipitation changes that vary synchronously with Greenland temperature changes (*5*). (**C**) Sinistral *Neogloboquadrina pachyderma* δ^18^O record at Orphan Knoll (core MD95-2024P), a proxy for freshwater flux into the North Atlantic (*27*, *28*). (**D**) Reconstructed ice thickness over the Rimrock Hills and Barnes Ice Cap margin (*35*). (**E**) Dates of Baffin Island calcite-forming events from this study and (*22*), traced with a probability density plot (PDP) calculated using (*76*). Bells indicate uncertainty distributions of the means (vertical lines) and heights scale with precision (only relative heights matter, absolute values omitted). (**F**) Carbonate ice-rafted detritus (IRD) and (**G**) water temperatures calculated from benthic foraminifera Mg/Ca (±1σ analytical uncertainty) at an intermediate-depth site in the Labrador Sea (core EW9302-2JPC) (*10*). (**H**) North Atlantic subsurface (∼150 m depth) water temperatures calculated from Mg/Ca ratios of sinistral *N. pachyderma* (envelope denotes 95% confidence interval) and (**I**) bulk sediment Ca/Sr, a proxy for carbonate IRD (core GeoB18530-1) (*14*). (**J**) Seawater δ^234^U (±2σ uncertainty) in the upper (<1.5 km depth) low-latitude North Atlantic Ocean, reconstructed from deep-sea corals (*19*).

We calculated U-Th dates of six subglacial carbonate precipitates from the BIC margin (*n* = 5; figs. S3 to S6) and Rimrock Hills region (*n* = 1; fig. S7 and see Supplementary Text). These dates record intermittent episodes of aqueous precipitation of calcite spanning 31 to 18 ka ago (Table 1 and Fig. 2E). Five samples record single episodes of subglacial calcite formation between 18 and 26 ka ago. One sample (M09-B176R) records multiple episodes of subglacial calcite formation and erosion at the BIC margin: a basal layer precipitated at ca. 31 ka ago and an upper layer that records calcite formation between 25 and 23 ka ago, with a >4.6-ka disconformity separating the two layers (Table 1 and fig. S6). The most precise calcite formation ages (±<1 ka at 95% confidence intervals) identify three episodes of subglacial carbonate formation on central Baffin Island at ca. 31, 25 to 23, and ca. 18.5 ka ago. Less precise ages overlap at least one of these episodes within 95% confidence intervals (Table 1). The time scales of carbonate formation we report are consistent with reliable U-Th dates reported by Refsnider *et al.* (*22*) for subglacial precipitates from the same localities (Table 1 and Supplementary Text).

**Table 1.** Model U-Th ages and initial δ^234^U (δ^234^_o_) compositions of central Baffin Island subglacial calcite-forming events. U All dates and δ^234^U_o_ from this study are reported with 95% confidence intervals, including systematic uncertainties from tracers and decay constants (*74*) and calculated relative to a 1950 CE datum. Lowercase letter and number designations for sample M09-B176R denote different layers of distinct age. See Supplementary Text and table S2 for further details and statistics. Dates and δ^234^U_o_ from Refsnider *et al.* (*22*) are reported as published with 2σ uncertainties (unspecified datum). We calculated the δ^234^U_o_ of M09-B064R (not reported previously) from the reported date and measured δ^234^U. See Supplementary Text for discussion on inclusion of previously published data.

**Sample**	**Model age (ka)**	**^δ234^U_o_ (‰)**
This study		
M09-B071R	18.38 ± 0.40	797.5 ± 7.1
M09-B184R	18.56 ± 0.58	1923 ± 71
M09-B152R	20.91 ± 3.68	2162 ± 93
M09-B183R	23.44 ± 4.14	1897.4 ± 38.6
M09-B177R	24.72 ± 1.54	1212.3 ± 6.6
M09-B176R.a1	23.43 ± 0.53	1559.6 ± 4.0
M09-B176R.a3	23.86 ± 0.49	1657.6 ± 2.8
M09-B176R.a4	24.32 ± 0.53	1736.0 ± 4.4
M09-B176R.a5	24.87 ± 0.29	1724.8 ± 3.3
M09-B176R.b	30.71 ± 0.89	1627.6 ± 8.0
Refsnider *et al.* (*22*)		
M09-B064R	19.1 ± 0.3	798 ± 11
M09-B153R	21.0 ± 5.4	2170 ± 260

Figure 2 compares the ages of these subglacial calcite-forming events with several paleoclimate proxies. Precise U-Th ages align closely with HEs, as recorded by several proxies (Fig. 2): extended (HE) stadials in the NGRIP δ^18^O record (*20*), HE-associated positive excursions of the Hulu cave δ^18^O record (*5*), and enhanced surface freshening in the North Atlantic recorded by planktic δ^18^O (*27*, *28*). Baffin Island calcite precipitation also coincides with the carbonate IRD peaks of H2 and H3 in two North Atlantic marine sediment cores (*10*, *14*). Both of these cores also record North Atlantic subsurface seawater temperatures calculated from the Mg/Ca ratios of foraminifera: one (*10*) at intermediate depths (1 to 2 km) overlapping the intervals of H1 and H3 (H2 occurs during a gap in the record) and the other (*14*) at shallower subsurface depths (∼150 m) over the last 27 ka, overlapping H1 and H2. Baffin Island subglacial calcite formation coincides with subsurface warming during H1 and H3 and immediately follows the subsurface warming preceding H2 (Fig. 2, E, G, and H). Apparent lags between the onset of detrital carbonate deposition in the western North Atlantic recorded in marine sediment cores EW9302-2JPC (Fig. 2F) and GeoB18530-1 (Fig. 2I) and the Hulu Cave speleothem-constrained onset of H2 and H3 may reflect a lag between HSIS iceberg flux and global climate response or a systematic offset in radiocarbon and U-Th chronologies. Regardless, the time frames of subglacial calcite formation on Baffin Island overlap with H2 and H3, whether measured by U-Th–constrained global climate response or radiocarbon-constrained IRD deposition (Fig. 2).

The various records indicate concordance between Baffin Island carbonate precipitation events and HEs, implying a relationship between HE processes and basal ice sheet processes over central Baffin Island when it was covered by the eastern flank of the Foxe Dome of the LIS. Precipitation of calcite requires liquid water at or above the saturation point with respect to calcite. Yet, ice sheet models predict persistent cold-based conditions across Baffin Island during the last glacial period (*24*), corroborated by geologic evidence of relatively nonerosive basal conditions (*29*). Given this regional distribution of cold-based conditions, the occurrence of subglacial precipitates across hundreds of meters at sites separated by tens of kilometers (*23*) and evidence for hydrologic connectivity to groundwaters from the Canadian interior (see the following sections) implies episodes of widespread basal melting over northeastern Baffin Island during HEs. The preservation of delicate surface features on the carbonates (fig. S1) confirms cold-based ice at other times, including the late Holocene. Glacial striae on the bedrock from which samples were collected and linear features on several of the samples themselves (fig. S1) indicate basal ice sliding in the direction of fjords that were occupied by ice streams at the time (*30*).

Our record requires that subglacial melting occurred beneath the northern LIS synchronously with the HSIS acceleration and iceberg discharge canonically associated with HEs. This synchrony strongly favors a shared climatic triggering mechanism to coordinate the response and is not consistent with a stimulus of internally paced binge/purge oscillations of ice stream surging (*8*). This assertion is a consequence of the importance of ice streams in regulating ice flow from the dome. In a modern ice sheet, changes in ice stream behavior can reconfigure local mass balance (*31*). Even under the binge/purge model (*8*), HSIS acceleration was controlled by thickening of the ice stream, not the tributary domes. Investigations of surging glaciers, representing a well-studied example of glacial oscillators, show that such glaciers do not go through synchronous cycles, even if they are proximal, close in size, and subject to similar climatic conditions (*32*). Our precipitate samples formed in the upstream regions of outlet glaciers that drained the Foxe Dome eastward through Scott Inlet and Buchan Gulf into Baffin Bay. These glaciological settings differ distinctly from the HSIS, which drained several domes (*15*) through a ≥100-km-wide trough, well over twice the size of the 10- to 40-km-wide main and tributary troughs of the Scott and Buchan ice streams (*30*, *33*). The most parsimonious explanation for the fact that these glacial features experienced simultaneous ice acceleration during H1, H2, and H3 (Fig. 2) is that they reacted to a common repeat climate forcing rather than that they experienced synchronous oscillations unrelated to climate changes.

**Fig. 3.**
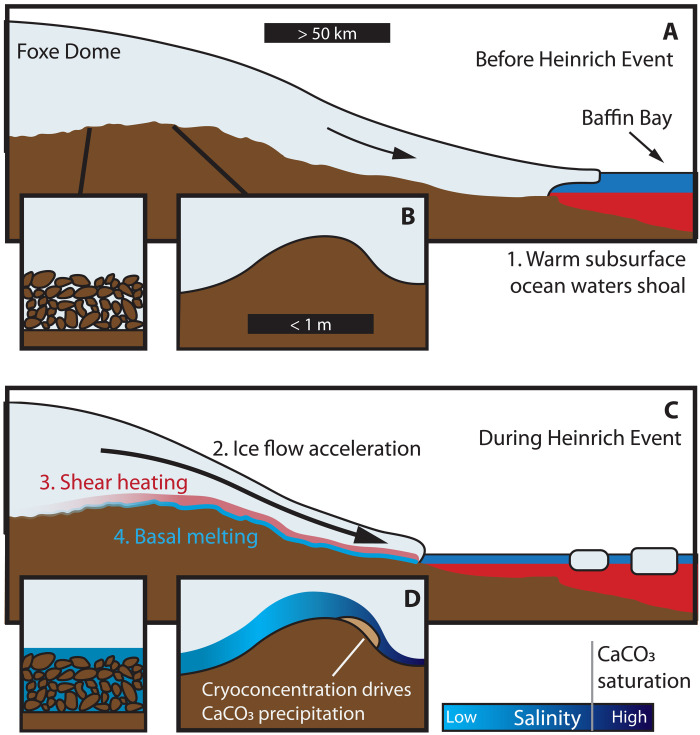
Schematic diagram summarizing the proposed model for calcite precipitation at the Barnes Ice Cap margin during HEs. Scale bars in (A) and (B) identify approximate horizontal length scales. Vertical dimension is not to scale. (**A** and **B**) Between HEs, the northeastern Laurentide Ice Sheet was frozen at the bed over Baffin Island and subglacial permafrost maintained low hydrologic connectivity. At the onset of HEs, warm subsurface waters from the North Atlantic shoaled in Baffin Bay and impinged on the grounding lines of ice streams that drained the Foxe Dome into Baffin Bay. (**C** and **D**) In response, these ice streams surged and accelerated ice flow over Baffin Island, resulting in shear heating and basal melting that enhanced subglacial hydrologic connectivity. As subglacial waters flowed over the location of the modern-day Barnes Ice Cap, localized refreezing in low-pressure zones on the lee sides of boulders and bedrock undulations cryoconcentrated residual waters to the point of calcite supersaturation and precipitation.

Alternatively, models relating HEs to subsurface ocean warming invoke HSIS surging in response to grounding line melting and retreat (*13*) or collapse of ice stream–buttressing ice shelves (*10*). Baffin Island ice streams that formerly occupied the Scott and Buchan Troughs, adjacent to the BIC, reached LGM grounding line extents >1000 m below modern-day sea level (*30*), the same intermediate water depths that record benthic warming correlated with HEs (*10*). We propose that basal melting and carbonate precipitation at the BIC margin occurred when Baffin Island ice streams surged in response to subsurface warming, accelerating inland ice velocities and elevating basal temperatures via shear heating (Fig. 3, Supplementary Text, and fig. S8). We estimate that ice acceleration initiated at Baffin Bay grounding lines took 250 to 650 years to propagate to the site of BIC calcite formation (Supplementary Text), a lag within the reported age uncertainties of calcite formation (Table 1) and consistent with the ca. 500-year duration of IRD deposition observed during HEs (*4*). Although the present chronologies lack the temporal resolution to evaluate the timing of subglacial calcite formation relative to the onset of HEs, both the Baffin Bay sedimentary record and patterns of deglacial sea level response on Baffin Island support the interpretation that the ice stream responses recorded by subglacial calcite formation were a primary response to subsurface ocean warming, rather than a secondary response to ocean-climate consequences of HEs (Supplementary Text). The enhanced ice flow velocity must be connected to Baffin Island ice stream surging rather than HSIS surging given the relative stability of the Foxe Dome during widespread LIS thinning associated with HSIS activity: The Keewatin and Quebec-Labrador Domes thinned substantially during the early deglacial period (20 to 15 ka ago), whereas the Foxe Dome remained relatively unchanged (Fig. 1D). Moreover, the time scales required to propagate thinning across the Foxe Dome ice divide to the site of calcite formation are too long to explain the tight coincidence of subglacial calcite formation with HEs (Supplementary Text).

Currently, the Baffin Island subglacial precipitate chronology does not identify any subglacial carbonate precipitation on Baffin Island preceding H3. One potential explanation is that reduced thickness of the LIS during MIS 3 (*34*, *35*) may have prevented Baffin Island ice streaming and basal melting during earlier HEs. Recuperated ice thickness at the BIC margin site by H3 (Fig. 2D) may have been necessary to sufficiently insulate the bed to accommodate melting during HE shear heating and/or to advance ice stream grounding lines to sufficient depth for exposure to subsurface warming events. Alternatively, limited preservation of pre-H3 carbonate precipitates may account for their absence in the present dataset. The disconformity in sample M09-B176R (Table 1 and fig. S6) confirms erosion between episodes of formation. Yet, the partial preservation of the basal layer implies that more ancient samples may have survived and might be identified by continued investigation of the BIC margin subglacial precipitate record.

Conversely, despite ongoing subsurface ocean warming during H1, subglacial calcite precipitation stopped by ca. 18 ka ago, before the onset of H1 IRD deposition (Fig. 2, E, F, and I). This may be an artifact of a small number of samples that fail to capture the full duration of calcite precipitation. The distinct morphological character, concordance of formation, and summit locations of the two 18- to 19-ka Rimrock Hills samples imply that their formation mechanism may be decoupled from the BIC margin precipitates and sensitive only to basal melting following the LGM. In either case, the only post-LGM BIC margin sample (M09-B184R; Table 1) predates the bulk of H1 iceberg discharge (*17*). While further investigation may reveal additional calcites formed across the H1 interval, we currently interpret the abbreviated H1 calcite precipitation to imply a shift in LIS dynamics and associated basal conditions during the glacial termination that began at this time (*36*).

### Subglacial fluid provenance

BIC margin and Rimrock Hills precipitates are restricted to the lee sides of bedrock undulations, bedrock fractures, and local depressions near summits, strongly supporting the conclusion that they form from the supersaturation of CaCO_3_, cryoconcentrated by the process of basal freezing in localized low-pressure zones (Fig. 3) (*22*, *23*). The extremely high U concentrations of the calcite (40 to >100 μg/g; data file S1) imply formation from a U-rich fluid, further supporting the role of cryoconcentration in increasing the ionic strength of the calcite-forming waters. Comparable calcite U concentrations are precedented in speleothems from permafrost environments (*37*), where cryoconcentration of source waters likely also occurs.

**Fig. 4.**
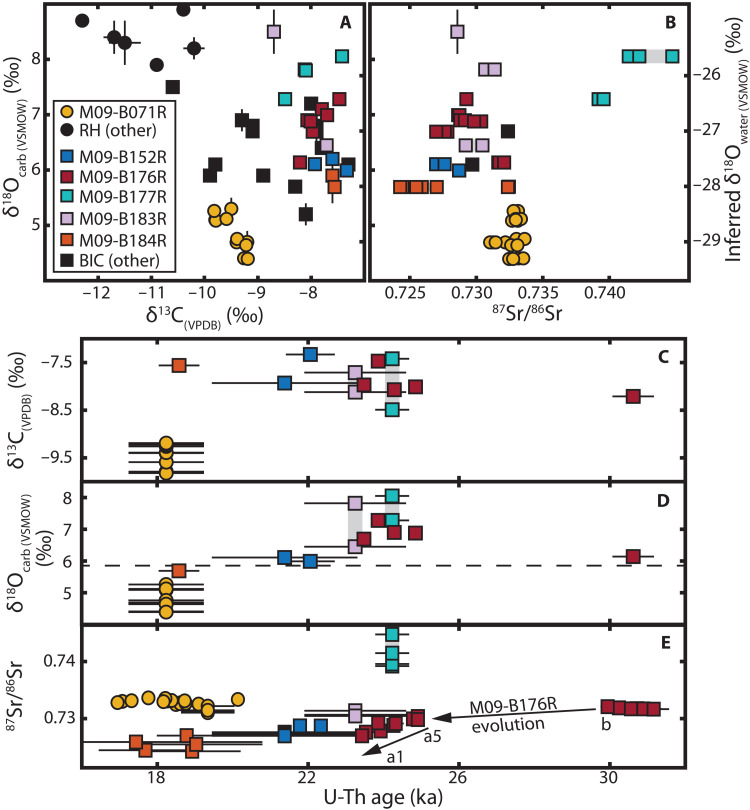
Carbonate δ^18^O, δ^13^C, and ^87^Sr/^86^Sr compositions of Baffin Island subglacial precipitates. Samples from Rimrock Hills (RH) denoted with circles and the Barnes Ice Cap (BIC) margin denoted with squares. (**A** and **B**) Carbonate compositions compiled from this study and (*23*). *y*-axis units reflect measured compositions (left) and compositions inferred for calcite-forming waters (right), assuming a water-carbonate fractionation of 33.6‰ at 0°C, after (*22*). RH samples other than M09-B071R plot beyond the bounds of (B) due to high ^87^Sr/^86^Sr (>0.77). (**C** to **E**) Carbonate δ^13^C, δ^18^O, and ^87^Sr/^86^Sr versus U-Th age. The dashed line in (D) represents the threshold of post-LGM δ^18^O compositions. Arrows in (E) indicate the stratigraphic progression of ^87^Sr/^86^Sr evolution in M09-B176R. ^87^Sr/^86^Sr uncertainties are 2σ SE. δ^13^C and δ^18^O uncertainties are 2σ SE of individual fractions (this study) or 1σ SD of replicate means (*23*). U-Th age error bars reflect 95% confidence intervals of analytical uncertainties. Gray bars indicate data that share a single age or isotope ratio due to different sampling resolutions for different methods.

Carbonate stable isotope compositions of δ^18^O and δ^13^C vary modestly (Fig. 4), consistent with the observations of Refsnider *et al.* (*23*). Our data reinforce their conclusions that δ^18^O reflects O isotopically fractionated during calcite precipitation from H_2_O in equilibrium with the overlying basal ice and δ^13^C reflects C isotopically fractionated from soil organic matter with minor contributions from bedrock calcite and atmospheric CO_2_. Over the course of >10 ka of intermittent calcite precipitation, δ^13^C does not change systematically with time, implying that this C source remained stable over this time frame (Fig. 4C). Similarly, ^87^Sr/^86^Sr does not vary systematically with time, except within sample M09-B176R, which records modest ^87^Sr/^86^Sr evolution during its growth (Fig. 4E). Overall, δ^18^O changes little with time, except for a slight shift from heavier compositions [δ^18^O_(VSMOW)_ > 5.9‰] preceding and during the LGM to lighter compositions (<5.8‰) after ca. 19 ka ago (Fig. 4), suggesting a shift toward isotopically lighter subglacial waters from melting of englacial ice (*23*) or delivery of late Pleistocene ice (*38*) to the bed. Together, these observations evidence limited secular change in the composition and provenance of subglacial waters at the BIC margin. Rather, variability in δ^13^C, δ^18^O, and ^87^Sr/^86^Sr compositions was controlled primarily by spatial heterogeneity in calcite-forming waters, and carbonate δ^18^O and δ^13^C compositions affirm the preponderance of basal meltwaters and soil-derived organic carbon as the respective sources of H_2_O and dissolved inorganic carbon (DIC) (*23*).

**Fig. 5.**
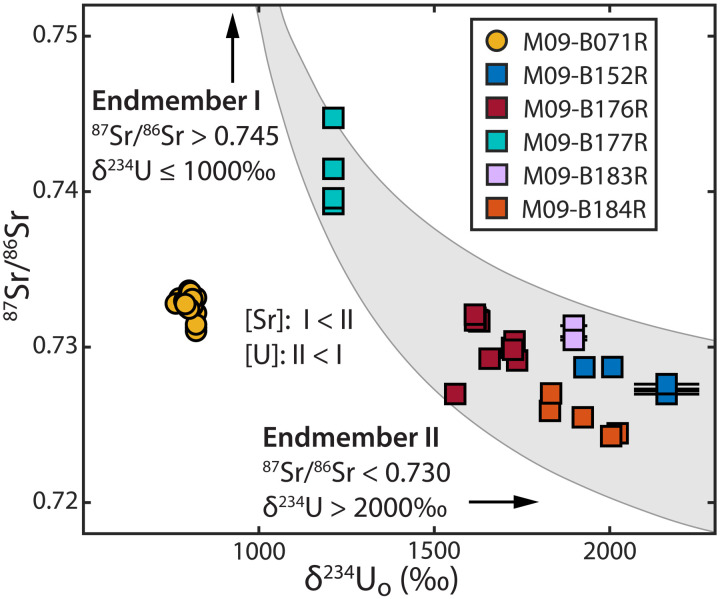
Paired initial δ^234^U (δ^234^U_o_) and ^87^Sr/^86^Sr compositions of subglacially formed carbonate precipitates from Baffin Island. Error bars denote 2σ SE, typically smaller than symbols. Shapes and colors as in Fig. 4. Individual fraction δ^234^U_o_ compositions are plotted to highlight the paired U-Sr isotopics of individual fractions. The isochron-calculated δ^234^U_o_ is used only where it differs significantly from the δ^234^U_o_ of its constituent fractions. The topology is consistent with a ≥2 endmember hyperbolic mixing model with endmember compositions indicated. To illustrate this relationship, the gray envelope demarcates a three-component admixture, after (*77*), assuming an endmember I–like composition (δ^234^U = 850‰, ^87^Sr/^86^Sr = 0.775), a range of plausible endmember II–like compositions (δ^234^U = 3000‰, 0.715≤^87^Sr/^86^Sr≤0.728), and relative elemental concentration ratios of [Sr]_I_/[Sr]_II_ = [U]_II_/[U]_I_ = 3.

Paired ^87^Sr/^86^Sr-δ^234^U data identify the presence of >2 distinct aqueous cation sources. Age-corrected initial δ^234^U (δ^234^U_o_) compositions are ∼798‰ at Rimrock Hills and range between 1200 and 2200‰ at the BIC margin, with no correlation between age and δ^234^U_o_ (Table 1). Paired ^87^Sr/^86^Sr-δ^234^U_o_ compositions of BIC margin samples lie along an approximately hyperbolic path reflecting isotopic mixture between ≥2 endmembers (Fig. 5). The heterogeneity in compositions distributed within a modeled three-component envelope requires >2 endmember waters or heterogeneity within “endmembers.” One minor component may be Sr and U leached from silicate detritus during carbonate digestion procedures. Even with these complexities, the ^87^Sr/^86^Sr-δ^234^U data provide constraints on the approximate isotopic compositions and relative concentrations of Sr and U in two primary endmembers, arbitrarily named I and II (Fig. 5).

The highly radiogenic ^87^Sr/^86^Sr signature of endmember I (Fig. 5) is consistent with local Baffin bedrock sources: glaciomarine sediments in the nearby Clyde Foreland range 0.74 ≤ ^87^Sr/^86^Sr ≤ 0.76, and Paleoproterozoic granites in southern Baffin Island (^87^Sr/^86^Sr > 0.78) are reasonable analogs for the felsic Precambrian basement rocks near the BIC margin and Rimrock Hills sites (*23*, *39*, *40*). In contrast, waters from more interior regions of central and northern continental Canada overlap the required ^87^Sr/^86^Sr < 0.73 of endmember II. Dissolved ^87^Sr/^86^Sr of rivers in the northern Hudson Bay region range from ∼0.72 to 0.73 (*41*). Deeply residing shield brines in Archaean to Proterozoic basement rocks of northern Canada and along the west coast of Hudson Bay range 0.710 < ^87^Sr/^86^Sr ≤ 0.728 (*42*). In addition, deep shield brines have higher total dissolved solids (TDS) than shallower groundwaters and converge on CaCl_2_ compositions (*43*), both of which may explain the higher Sr content of endmember II than endmember I.

We are not aware of modern aqueous δ^234^U measurements on Baffin Island, but the elevated δ^234^U_o_ compositions of both endmembers provide robust evidence for groundwater provenance (Fig. 5). Elevated aqueous δ^234^U is associated with the time-dependent ejection of ^234^Th, which rapidly decays to ^234^U, from sediment surfaces following α-decay of ^238^U. Aqueous ^234^U enrichment in sediment/rock pore space scales with increased residence time, lower porosity, and lower aqueous U concentration (*44*). Thus, while δ^234^U > 0 is typical among terrestrial waters, the largest enrichments are observed in groundwaters (*45*), with deep groundwater δ^234^U compositions commonly several thousand per mille (*46*). Waters with δ^234^U > 2000‰, as required by endmember II, are found in both bedrock aquifers (*44*, *46*) and groundwater-permafrost systems (*37*, *47*). We expect permafrost to have similar or elevated δ^234^U compared to liquid groundwaters of similar depth, as permafrost and slow-flowing subpermafrost groundwaters can experience protracted residence in contact with rock and sediment. Moreover, permafrost efficiently fractures rock (*48*), which increases surface area and enhances ^234^U injection, a process that may have been augmented by subglacial hydraulic pressures from the LIS (*49*). Thus, we conclude that subglacial groundwater aquifers are the most probable source of the inferred high-δ^234^U waters recorded by the calcites from Baffin Island. Since deeper-residing groundwaters exhibit higher δ^234^U than their shallower counterparts by as much as 1000‰ or more (*44*, *50*), we identify the lower-δ^234^U endmember I as a relatively shallow groundwater and the higher-δ^234^U endmember II as a more deeply derived groundwater, consistent with the respective provenances inferred from Sr isotopes.

Stable isotope compositions do not vary systematically with ^87^Sr/^86^Sr (Fig. 4), indicating that mixing groundwater sources had little to no effect on O and C systematics. The most parsimonious explanation is that the local subglacial meltwater was initially well mixed with shallow Baffin Island groundwater (endmember I) and was the volumetrically dominant component of the calcite-forming waters. Sr and U reflect admixture with small volumes of high-TDS groundwaters (endmember II) that left isotopic compositions of H_2_O and DIC unperturbed. Even if larger volumes were incorporated, the effect on δ^13^C and δ^18^O would be minor, since deep saline and brine groundwaters have low DIC contents (*51*) and δ^18^O similar to or lighter than local meteoric water: Saline groundwaters in northern central Canada (*52*) approach the δ^18^O of BIC basal ice (*22*).

In summary, between 31 and 18 ka ago, Baffin Island shallow groundwaters were well mixed with LIS basal meltwater (in equilibrium with the basal ice) and had DIC predominantly derived from oxidized soil organic matter. These shallow groundwaters mixed with small volumes of saline or briny groundwaters distally sourced from deep bedrock aquifers toward the continental interior, altering cation compositions but having no measurable effect on the isotopic composition of H_2_O or DIC. Unlike the BIC margin site, the Rimrock Hills sites were isolated from distally sourced (endmember II) groundwaters, as evidenced by δ^234^U_o_<800‰ and inconsistency with the BIC margin mixing relationship (Fig. 5), perhaps due to their locations at local summits (*22*, *39*) and other regional factors that limited groundwater influx.

## DISCUSSION

### Subglacial groundwaters of the LIS

Precipitates at the modern-day BIC margin record the presence of waters from 31 to 18 ka ago that are compositionally consistent with high-salinity groundwaters found at depths of tens to hundreds of meters in crystalline basement rocks of the Canadian shield (*42*, *44*, *51*). The presence of these high-density shield brines at the base of the LIS requires a powerful physical mechanism to transport them to the subglacial surface. We propose that this was achieved by glaciohydraulic reorganization of groundwater flow beneath the LIS. The overlying weight of ice sheets drove infiltration of basal meltwaters beneath the ice sheet interior, pressurizing subglacial aquifers and generating a steep hydraulic gradient between the ice sheet interior and margin (*53*, *54*). Fresh meltwaters were driven deep into the subsurface and mixed with high-TDS shield brines, as evidenced by geochemical observations of Canadian groundwaters at depths up to 1300 m (*54*, *55*) and numerical simulations of LIS groundwater recharge (*56*). As the water flowed along hydraulic gradients to the ice sheet margin, the flow paths shallowed and brought deeply sourced shield brines toward the surface. Simulations of sub-LIS groundwater flow predict upwelling of these brines extending ∼100 km interior from the northeastern LIS margin (*56*). Despite predicted permafrost conditions at the BIC margin over this time frame (*24*, *56*, *57*), aqueous mixing (Fig. 5) and calcite precipitation required intermittent subglacial permafrost melting and connectivity with deep groundwaters (Fig. 3), perhaps facilitated by fracture networks and freezing-point depression from the introduction of high-salinity brines. Moreover, the time-dependent evolution of ^87^Sr/^86^Sr in M09-B176R (Fig. 4) suggests that this hydrologic connectivity supported ongoing subglacial fluid transport over the course of basal melting events.

In addition to the Baffin Island record, elevated δ^234^U compositions are found widely in marginal zones of the LGM LIS (Fig. 1A). Speleothems from the southern LIS margin and within 20 to 50 m of the modern surface record δ^234^U_o_ ranging from 800 to 8000‰, with the majority >2000‰ over the last 250 ka (*47*). Late Pleistocene speleothems from caves near the southeastern LIS margin record δ^234^U_o_ compositions in excess of 1000‰ (*58*). Cryogenically precipitated carbonates from LIS basal waters near the northwestern margin record δ^234^U_o_∼800‰ at ca. 19 ka ago (*59*). Speleothems in the southern Canadian Rockies record δ^234^U_o_ in excess of 1000‰ (*60*). The high-δ^234^U source waters may have been either sourced from deep groundwaters or evolved locally through protracted residence of porewater in permafrost environments (*61*). Permafrost provenance is less probable at low elevations since groundwater discharge during glacial terminations (*57*) would have interrupted the requisite porewater residence required for substantial in situ ^234^U enrichment (*61*). Nonetheless, the groundwater-permafrost distinction is functionally unimportant: The mechanisms of ^234^U-enrichment by α-recoil injection are identical in bedrock aquifer, subglacial groundwater, and permafrost settings and all produce high-δ^234^U subglacial and periglacial groundwaters (*44*, *61*, *62*).

Collectively, subglacial precipitates on Baffin Island and marginal speleothems and cryogenic carbonates from around the LIS formed discontinuously in time, indicating that melting events at all of these marginal sites were typically transient or episodic. Although widespread subglacial hydrologic connectivity brought deep interior waters toward the LIS margins, the margins themselves were typically cold-based with robust marginal permafrost systems. These subsolidus marginal conditions not only restricted the growth of carbonate precipitates to critical intervals of climatic and glaciologic activity but also retained groundwaters within the ice sheet margins during glacial conditions.

### Subglacial groundwaters and early deglacial ocean uranium chemistry

Records of high-δ^234^U shallow and surfacing groundwaters encircling the LIS margin (Fig. 1) imply that such waters were commonplace, constituting a substantial reservoir of continental waters with elevated δ^234^U stored in permafrost and (potentially pressurized) subglacial aquifers (*53*). However, extensive subglacial melting could breach these confining cold-based margins and marginal permafrosts systems and efficiently route the high-δ^234^U waters to the margin. Although the areal extent of the LIS remained stable from the LGM until ca. 15 ka ago (*1*) and ice sheet models predict that LGM-like ice volumes persisted up to this time, areal basal melt increased from <40 to >60% between 25 and 15 ka ago (*24*), providing an efficient mechanism to melt permafrost, enhance hydrologic connectivity, and deliver interior high-δ^234^U waters to the ice sheet margin and ultimately the ocean.

Such a scenario of subglacial drainage would account for the abbreviated duration of H1 subglacial calcite formation. High-δ^234^U calcite-forming subglacial waters were present near the modern BIC margin during three consecutive HEs, but carbonate precipitation ended prematurely during H1, just as Atlantic δ^234^U compositions were rising toward their deglacial peak (Fig. 2, E and J). Before ca. 18 ka ago, we propose that calcite-forming waters were restrained by cold-based margins and continuous marginal permafrosts that persisted during nonterminal HEs. Then, during extended H1-deglacial conditions, enhanced LIS basal melting and hydrologic connectivity (*24*) breached these marginal features and routed the high-δ^234^U calcite-forming waters to the ice sheet margin, depleting the subglacial reservoir and shutting down carbonate growth by ca. 17 ka ago.

Therefore, we identify LIS groundwaters, broadly encompassing liquid porewaters in subglacial tills as well as permafrost in sub- and proglacial settings, as a compelling candidate for the subglacial reservoir responsible for the 6‰ enrichment of Atlantic Ocean δ^234^U during early deglaciation (*19*). Chen *et al.* (*19*) simulated the upper North Atlantic record by reducing a simplified model AMOC by 50% and enhancing the δ^234^U of modern riverine and groundwater inputs into the upper Atlantic from 339 to 800‰. In comparison, the δ^234^U of LIS-associated groundwaters considered here typically exceed 800‰. Subglacial precipitates do not reliably record the U concentration of their parent groundwaters; however, deep groundwaters with elevated δ^234^U are typically >10 μg U/liter (*46*) and Arctic permafrosts with δ^234^U approaching 1000‰ range from ∼1 to 50 μg/liter (*61*). Both exceed the U content of seawater (∼3 μg/liter) (*63*), indicating that subglacial groundwaters and melted permafrost may serve as powerful levers on ocean δ^234^U at lesser volumes than riverine input (typically ≪3 μg/liter) (*45*).

Marine and terrestrial records indicate that large-scale release of ^234^U-rich subglacial fluids into the North Atlantic was apparently restricted to H1 and the early deglacial period. While H1 bore many of the hallmarks of preceding HEs, this ultimate iceberg discharge event was unique in both its evacuation of subglacial aquifers and its concurrence with termination of the last glacial period, implying a potential mechanistic relationship between these events (*18*, *64*). We speculate that before the LGM, marginal cold-based conditions and permafrost systems effectively restrained these interior subglacial aquifers. Following the LGM, extensive subglacial melting (*24*) enhanced hydrologic connectivity and degraded marginal permafrost systems, releasing subglacial waters that ultimately drained into the ocean. The concurrence of this process with H1 iceberg discharge implies that post-LGM subglacial melting promoted iceberg flux, HE-associated ice stream acceleration promoted extensive basal melting, or both processes reinforced each other. For now, the evidence is still limited and motivates further investigation of both subglacial and farther-afield records to examine the relationships among H1, the last glacial termination, and the role of subglacial hydrology in both.

### Recontextualizing HEs

Subglacial calcites at the BIC margin formed during LIS basal melting events caused by nearby ice stream acceleration and implicate subsurface North Atlantic warming as a causal mechanism of HEs (Fig. 3), independent of traditional HSIS evidences. Between HEs, the grounding lines of these ice stream systems readvanced to intermediate ocean water depths, requiring a rethickening of ice over Baffin Island, just as in the Hudson Strait. Although we show that ocean forcing initiated HEs, the recovery and regrowth phase, as in (*8*), remains a crucial component of the cycle. Notably, the coordinated ice stream response implies that Baffin ice streams and the HSIS recovered over similar time frames, potentially coupled via far-reaching isostatic adjustment from eastern LIS domes (*13*) or rethickening of the shared Foxe Dome. Evidence for persistent open-water and sea-ice conditions over Baffin Bay (*11*) emphasizes the central role of ice streams, rather than extensive ice shelves, in controlling HE responses to subsurface ocean warming, though minor ice shelves and sea ice may have modulated these ice stream processes, e.g., via buttressing (*10*, *65*).

The climatic sensitivity of marine-terminating outlet glaciers on Baffin Island is supported by evidence of fluctuating sediment delivery from eastern Baffin Island into Baffin Bay on D-O time scales (*66*, *67*). While these sedimentary archives record outlet glacier responses to millennial-scale ocean-climate forcings, our data show that only HE conditions were sufficient to trigger an interior ice sheet response. Subglacial calcite forming events occurred on the interior highlands of Baffin Island, >600 m above modern sea level and >180 km from the LGM ice stream grounding lines. Since ice acceleration in response to grounding line retreat propagated at least this far into the ice sheet interior, this process reflects a regional, ice sheet–scale response to ocean forcing during HEs (Fig. 3). Yet, these ice sheet–scale episodes of acceleration and subglacial melting over Baffin Island were transient conditions: aqueous precipitates and nearby striated bedrock (*23*) require episodes of warm-based ice conditions, but inherited cosmogenic radionuclide signatures within the striated bedrock require a persistent state of cold-based, nonerosive conditions (*68*). To satisfy these observations, HE ice acceleration over Baffin Island must have reflected relatively brief (e.g., <1 ka) episodes of basal melting superimposed over the long-term cold-based conditions. The only sample that precisely records time across a single HE, the upper layer of sample M09-B176R (layers a1 to a5; Table 1), formed during H2 in as little as 800 years, corroborating the predicted brevity of melting episodes over the interior of Baffin Island. Substantial ice thicknesses over the region, ranging from ∼1.5 km at the site of calcite formation (Fig. 2D) to >3 km at the Foxe Dome (*35*), may have provided steep ice surface slopes that supported this rapid and extensive ice acceleration.

The rapid, coordinated regional response of ice streams terminating in both the Labrador Sea and Baffin Bay suggests that other maritime sectors of the eastern LIS, and perhaps other ice sheets, may have responded similarly to ocean forcing during HEs. Widespread subsurface ocean warming and subsequent surging of various marine-terminating ice streams in the North Atlantic would account for the heterogeneity in IRD provenance observed in HE layers, which suggests iceberg flux from a variety of sources including Baffin Island, Greenland, Iceland, and Eurasian ice sheets (*4*, *69*). Such ocean-coordinated ice sheet responses across the North Atlantic provide a framework to interpret the relationship between HEs and their northern Pacific analog, Siku events—massive iceberg discharge episodes of the Cordilleran Ice Sheet that precede HEs (*70*). On the basis of our findings of coordinated ice stream acceleration during HEs, we speculate that episodes of ice sheet–wide ice stream activation reflect intra-oceanic responses to millenial-scale interoceanic climate oscillations.

## MATERIALS AND METHODS

We studied six subglacially formed detritus-rich carbonate precipitate rocks, including five samples from the northern BIC margin and one sample from the Rimrock Hills region (Fig. 1 and fig. S1), originally reported by Refsnider *et al.* (*22*, *23*). Small “crust” samples (M09-B184R and M09-B152) were subsampled with steel hand tools, while larger samples were slabbed and subsampled with a rock saw before isolating individual fractions with hand tools.

### Carbon and oxygen isotope measurements

Isotopes of oxygen and carbon in carbonate phases were measured at the UC Santa Cruz Stable Isotopes Laboratory by acid digestion using an individual vial acid drop Thermo Fisher Scientific Kiel IV carbonate device interfaced to a Thermo Fisher Scientific MAT 253 dual-inlet isotope ratio mass spectrometer (iRMS). We loaded ∼100-μg fractions into individual vials that were dried overnight in a 70°C vacuum oven. Samples reacted at 75°C with H_3_PO_4_ (1.92 g/cm^3^ specific gravity) to generate CO_2_ and H_2_O. The latter is cryogenically separated, and noncondensible gases are pumped away before introduction of the CO_2_ analyte into the iRMS. Samples are measured concurrently with replicates of NBS-18 limestone standard reference material and a Carrara Marble in-house standard (CM12, calibrated to NBS-18 and NBS-19). Carbonate δ^18^O and δ^13^C values are calculated relative to Vienna PeeDee Belemnite (VPDB) with a two-point calibration between CM12 and NBS-18 to correct for offset and linearity. Reproducibility and independent quality control are monitored with measurements of “Atlantis II” powdered coral. We convert δ^18^O_(VPDB)_ to δ^18^O_(VSMOW)_ with δ^18^O_(VSMOW)_ = 1.03091 × δ^18^O_(VPDB)_ + 30.91, after (*71*).

### Uranium, thorium, and strontium isotope measurements

Individual carbonate precipitate fractions were cleaned by sonication at room temperature in methanol for 30 min, triple-rinsed with ultrapure water (deionized to 18 megohms·cm), and transferred to an acid-cleaned perfluoroalkoxy alkane (PFA) beaker. All aqueous reagents (except ultrapure water) were either triple-distilled or commercial trace-metal grade. We submerged cleaned samples in approximately 1 ml of ultrapure water and added 7 M HNO_3_ dropwise to gently digest the carbonate minerals. When the reaction subsided, we brought the solution to 3 M HNO_3_, spiked with a gravimetrically calibrated mixed ^229^Th-^236^U tracer, and warmed the solution for several hours to progress reactions to completion. We twice evaporated the fractions to dryness, rehydrated in 7 M HNO_3_, and refluxed at 110°C to ensure complete digestion and sample-spike equilibration.

We purified Th and U separates from each dissolved fraction using ion chromatography on a 1-ml bed of AG1-X8 anion-exchange resin (200 to 400 mesh). We introduced dissolved samples onto pre-cleaned resin in 1 ml of 7 M HNO_3_ and eluted matrix ions (including Sr) with 2 ml of 7 M HNO_3_ followed by 250 μl of 6 M HCl. We eluted Th in 2 ml of 6 M HCl followed by U in 3 ml of water into a single beaker. We twice evaporated the U-Th separate to dryness and rehydrated with 7 M HNO_3_ to convert to nitrate salts. We then repeated the same ion chromatography procedure and collected the Th and U elutions separately. We evaporated the elutions just to dryness, refluxed at 110°C in 250 μl of 30% H_2_O_2_ for several hours to remove organic compounds, and lastly dried the solutions with trace H_3_PO_4_. Total procedural blanks of Th and U were <50 and <80 pg, respectively, and negligible compared to sample sizes (hundreds of nanograms of U and ≫10 ng of Th).

To purify Sr, we evaporated matrix elutions from the primary U-Th column to dryness, rehydrated in 0.5 ml of 7 M HNO_3_, and introduced this solution onto a 0.5-ml bed of precleaned Sr-Spec resin. We eluted matrix with 3 ml of 7 M HNO_3_ and collected purified Sr in 4 ml of 0.05 M HNO_3_. We dried the latter with trace H_3_PO_4_.

We measured isotopes of U, Th, and Sr on the Isotopx X62 thermal ionization mass spectrometer at UC Santa Cruz. All samples were loaded onto degassed 99.99% purity Re ribbons. We loaded U with a Si gel–0.035 M H_3_PO_4_ activator and measured UO_2_ isotopologs using a dynamic Faraday-Daly method (*62*). We calculated U isotope compositions from UO_2_ isotopologs by correcting for oxide isobaric interferences, although these corrections were negligible compared to analytical uncertainties. We corrected for mass-dependent fractionation with a linear model calibrated from long-term standard measurements and calibrated the photomultiplier deadtime from measurements of NBS SRM U-500 (as UO_2_). The accuracy of U isotope measurements were validated over the course of this study with replicate measurements of National Institute of Standards and Technology Standard Reference Material (NIST SRM) 4321b (fig. S2). We loaded Th with 1 μl of 5% HNO_3_ onto Re filaments coated with graphite and measured isotopes of Th as a metal on the Daly-photomultiplier complex using a peak hopping method. Th isotope ratios were corrected for mass-dependent fractionation and photomultiplier deadtime using model values determined by measurements of SRM U-500 ionized as a metal.

U-Th isotope measurements were spike-subtracted with an algorithm that fully propagates analytical and tracer uncertainties, assuming uncorrelated uncertainties. We report and interpret measured isotopic data as activity ratios, denoted with parentheses. In the case of U isotopes, we report carbonate U compositions as activity ratios and report inferred calcite-forming water compositions in δ notation. To precisely calculate U-Th dates and δ^234^U_o_, we used a Monte Carlo method algorithm (10^6^ trials per fraction) that fully propagates the uncertainties of all input isotopic ratios, including corrections for detrital contributions where necessary. Dates are calculated relative to a 1950 CE “present” datum. The accuracy of U-Th dates and δ^234^U_o_ was confirmed with concurrent measurement of a Marine Isotope Stage 5e coral (data file S1).

We loaded Sr with a TaCl_5_ activator and measured Sr isotopes on Faraday cups with a static collection method. We correct for isobaric interference from ^87^Rb on ^87^Sr by concurrent measurement of ^85^Rb and subtracting the intensity scaled by an assumed ^87^Rb/^85^Rb = 0.386. We calculate a mass-dependent fractionation β factor from the measured ^86^Sr/^88^Sr with an exponential law, assuming a canonical ^86^Sr/^88^Sr = 0.11940. We calculate fully corrected radiogenic ^87^Sr/^86^Sr compositions by applying this β factor to ^87^Rb-corrected ^87^Sr/^86^Sr ratios. Before data collection, we confirmed reproduction of NIST SRM 987: ^87^Sr/^86^Sr = 0.710250 ± 0.000011 (1σ SD) (*72*). Over the course of this study, we report a mean SRM 987 ^87^Sr/^86^Sr = 0.710232 ± 0.000027 (2σ SE, *n* = 8), consistent with the accepted value. Given the detritus-rich nature of the carbonate rock samples, we consider the U-Th-Sr isotope systematics in detail and quantify radiogenic and detrital components with isochron-based methods to calculate U-Th dates (Supplementary Text and figs. S3 to S7).

## Supplementary Materials

### This PDF file includes:

Supplementary Text Figs. S1 to S8 Tables S1 to S3 References

## References

[1] A. S. Dyke, J. T. Andrews, P. U. Clark, J. H. England, G. H. Miller, J. Shaw, J. J. Veillette, The Laurentide and Innuitian ice sheets during the Last Glacial Maximum. Quat. Sci. Rev. 21, 9–31 (2002).

[2] P. U. Clark, A. S. Dyke, J. D. Shakun, A. E. Carlson, J. Clark, B. Wohlfarth, J. X. Mitrovica, S. W. Hostetler, A. M. McCabe, The Last Glacial Maximum. Science 325, 710–714 (2009). 19661421 10.1126/science.1172873

[3] L. C. Menviel, L. C. Skinner, L. Tarasov, P. C. Tzedakis, An ice–climate oscillatory framework for Dansgaard–Oeschger cycles. Nat. Rev. Earth Environ. 1, 677–693 (2020).

[4] S. R. Hemming, Heinrich events: Massive late Pleistocene detritus layers of the North Atlantic and their global climate imprint. Rev. Geophys. 42, RG1005 (2004).

[5] Y. J. Wang, H. Cheng, R. L. Edwards, Z. S. An, J. Y. Wu, C.-C. Shen, J. A. Dorale, A high-resolution absolute-dated Late Pleistocene monsoon record from Hulu Cave, China. Science 294, 2345–2348 (2001). 11743199 10.1126/science.1064618

[6] W. F. Ruddiman, Late Quaternary deposition of ice-rafted sand in the subpolar North Atlantic (lat 40° to 65°N). GSA Bulletin 88, 1813–1827 (1977).

[7] H. Heinrich, Origin and consequences of cyclic ice rafting in the Northeast Atlantic Ocean during the past 130,000 years. Quatern. Res. 29, 142–152 (1988).

[8] D. R. MacAyeal, Binge/purge oscillations of the Laurentide Ice Sheet as a cause of the North Atlantic’s Heinrich events. Paleoceanography 8, 775–784 (1993).

[9] R. Zahn, J. Schönfeld, H.-R. Kudrass, M.-H. Park, H. Erlenkeuser, P. Grootes, Thermohaline instability in the North Atlantic during meltwater events: Stable isotope and ice-rafted detritus records from Core SO75-26KL, Portuguese Margin. Paleoceanogr. Paleoclimatol. 12, 696–710 (1997).

[10] S. A. Marcott, P. U. Clark, L. Padman, G. P. Klinkhammer, S. R. Springer, Z. Liu, B. L. Otto-Bliesner, A. E. Carlson, A. Ungerer, J. Padman, F. He, J. Cheng, A. Schmittner, Ice-shelf collapse from subsurface warming as a trigger for Heinrich events. Proc. Natl. Acad. Sci. U.S.A. 108, 13415–13419 (2011). 21808034 10.1073/pnas.1104772108 PMC3158189

[11] A. E. Jennings, J. T. Andrews, C. Ó Cofaigh, G. St-Onge, S. Belt, P. Cabedo-Sanz, C. Pearce, C. Hillaire-Marcel, D. Calvin, Baffin Bay paleoenvironments in the LGM and HS1: Resolving the ice-shelf question. Mar. Geol. 402, 5–16 (2018).

[12] R. Hesse, I. Klauck, S. Khodabakhsh, D. Piper, Continental slope sedimentation adjacent to an ice margin. III. The upper Labrador Slope. Mar. Geol. 155, 249–276 (1999).

[13] J. N. Bassis, S. V. Petersen, L. Mac Cathles, Heinrich events triggered by ocean forcing and modulated by isostatic adjustment. Nature 542, 332–334 (2017). 28202970 10.1038/nature21069

[14] L. Max, D. Nürnberg, C. M. Chiessi, M. M. Lenz, S. Mulitza, Subsurface ocean warming preceded Heinrich Events. Nat. Commun. 13, 4217 (2022). 35864111 10.1038/s41467-022-31754-x PMC9304376

[15] F. A. Ziemen, M.-L. Kapsch, M. Klockmann, U. Mikolajewicz, Heinrich events show two-stage climate response in transient glacial simulations. Clim. Past 15, 153–168 (2019).

[16] D. Roche, D. Paillard, E. Cortijo, Constraints on the duration and freshwater release of Heinrich event 4 through isotope modelling. Nature 432, 379–382 (2004). 15549102 10.1038/nature03059

[17] J. D. Stanford, E. J. Rohling, S. Bacon, A. P. Roberts, F. E. Grousset, M. Bolshaw, A new concept for the paleoceanographic evolution of Heinrich event 1 in the North Atlantic. Quat. Sci. Rev. 30, 1047–1066 (2011).

[18] D. A. Hodell, J. A. Nicholl, T. R. R. Bontognali, S. Danino, J. Dorador, J. A. Dowdeswell, J. Einsle, H. Kuhlmann, B. Martrat, M. J. Mleneck-Vautravers, F. J. Rodríguez-Tovar, U. Röhl, Anatomy of Heinrich Layer 1 and its role in the last deglaciation. Paleoceanography 32, 284–303 (2017).

[19] T. Chen, L. F. Robinson, M. P. Beasley, L. M. Claxton, M. B. Andersen, L. J. Gregoire, J. Wadham, D. J. Fornari, K. S. Harpp, Ocean mixing and ice-sheet control of seawater ^234^U/^238^U during the last deglaciation. Science 354, 626–629 (2016). 27811276 10.1126/science.aag1015

[20] I. K. Seierstad, P. M. Abbott, M. Bigler, T. Blunier, A. J. Bourne, E. Brook, S. L. Buchardt, C. Buizert, H. B. Clausen, E. Cook, D. Dahl-Jensen, S. M. Davies, M. Guillevic, S. J. Johnsen, D. S. Pedersen, T. J. Popp, S. O. Rasmussen, J. P. Severinghaus, A. Svensson, B. M. Vinther, Consistently dated records from the Greenland GRIP, GISP2 and NGRIP ice cores for the past 104 ka reveal regional millennial-scale δ^18^O gradients with possible Heinrich event imprint. Quat. Sci. Rev. 106, 29–46 (2014).

[21] M. Margold, C. R. Stokes, C. D. Clark, Ice streams in the Laurentide Ice Sheet: Identification, characteristics and comparison to modern ice sheets. Earth Sci. Rev. 143, 117–146 (2015).

[22] K. A. Refsnider, G. H. Miller, C. Hillaire-Marcel, M. L. Fogel, B. Ghaleb, R. Bowden, Subglacial carbonates constrain basal conditions and oxygen isotopic composition of the Laurentide Ice Sheet over Arctic Canada. Geology 40, 135–138 (2012).

[23] K. A. Refsnider, G. H. Miller, M. L. Fogel, B. Fréchette, R. Bowden, J. T. Andrews, G. L. Farmer, Subglacially precipitated carbonates record geochemical interactions and pollen preservation at the base of the Laurentide Ice Sheet on central Baffin Island, eastern Canadian Arctic. Quatern. Res. 81, 94–105 (2014).

[24] S. J. Marshall, P. U. Clark, Basal temperature evolution of North American ice sheets and implications for the 100-kyr cycle. Geophys. Res. Lett. 29, 67-1–67-4 (2002).

[25] G. K. C. Clarke, U. Nitsan, W. S. B. Paterson, Strain heating and creep instability in glaciers and ice sheets. Rev. Geophys. 15, 235–247 (1977).

[26] C. Ritz, Time dependent boundary conditions for calculating of temperature fields in ice sheets, in *The Physical Basis of Ice Sheet Modeling* (International Association of Hydrological Sciences, 1987), pp. 207–216.

[27] C. Hillaire-Marcel, G. Bilodeau, Instabilities in the Labrador Sea water mass structure during the last climatic cycle. Can. J. Earth Sci. 37, 795–809 (2000).

[28] J. Lynch-Stieglitz, M. W. Schmidt, L. Gene Henry, W. B. Curry, L. C. Skinner, S. Mulitza, R. Zhang, P. Chang, Muted change in Atlantic overturning circulation over some glacial-aged Heinrich events. Nat. Geosci. 7, 144–150 (2014).

[29] D. E. Sugden, Glacial erosion by the Laurentide Ice Sheet. J. Glaciology 20, 367–391 (1978).

[30] E. Brouard, P. Lajeunesse, Maximum extent and decay of the Laurentide Ice Sheet in Western Baffin Bay during the Last glacial episode. Sci. Rep. 7, 10711 (2017). 28878304 10.1038/s41598-017-11010-9 PMC5587637

[31] I. Joughin, S. Tulaczyk, Positive mass balance of the Ross Ice Streams, West Antarctica. Science 295, 476–480 (2002). 11799237 10.1126/science.1066875

[32] D. I. Benn, A. C. Fowler, I. Hewitt, H. Sevestre, A general theory of glacier surges. J. Glaciol. 65, 701–716 (2019).

[33] M. Margold, C. R. Stokes, C. D. Clark, J. Kleman, Ice streams in the Laurentide Ice Sheet: A new mapping inventory. J. Maps 11, 380–395 (2015).

[34] A. S. Dalton, S. A. Finkelstein, S. L. Forman, P. J. Barnett, T. Pico, J. X. Mitrovica, Was the Laurentide Ice Sheet significantly reduced during Marine Isotope Stage 3? Geology 47, 111–114 (2019).

[35] E. J. Gowan, X. Zhang, S. Khosravi, A. Rovere, P. Stocchi, A. L. C. Hughes, R. Gyllencreutz, J. Mangerud, J.-I. Svendsen, G. Lohmann, A new global ice sheet reconstruction for the past 80 000 years. Nat. Commun. 12, 1199 (2021). 33623046 10.1038/s41467-021-21469-w PMC7902671

[36] G. H. Denton, R. F. Anderson, J. R. Toggweiler, R. L. Edwards, J. M. Schaefer, A. E. Putnam, The last glacial termination. Science 328, 1652–1656 (2010). 20576882 10.1126/science.1184119

[37] A. Vaks, O. S. Gutareva, S. F. M. Breitenbach, E. Avirmed, A. J. Mason, A. L. Thomas, A. V. Osinzev, A. M. Kononov, G. M. Henderson, Speleothems reveal 500,000-year history of Siberian permafrost. Science 340, 183–186 (2013). 23429705 10.1126/science.1228729

[38] A. C. Mix, W. F. Ruddiman, Oxygen-isotope analyses and Pleistocene ice volumes. Quatern. Res. 21, 1–20 (1984).

[39] J. T. Andrews, J. D. Ives, G. K. Guennel, J. L. Wray, An Early Tertiary outcrop in North-Central Baffin Island, Northwest Territories, Canada: Environment and significance. Can. J. Earth Sci. 9, 233–238 (1972).

[40] D. Lacelle, B. Lauriol, I. D. Clark, Origin, age, and paleoenvironmental significance of carbonate precipitates from a granitic environment, Akshayuk Pass, southern Baffin Island, Canada. Can. J. Earth Sci. 44, 61–79 (2007).

[41] M. A. Wadleigh, J. Veizer, C. Brooks, Strontium and its isotopes in Canadian rivers: Fluxes and global implications. Geochim. Cosmochim. Acta 49, 1727–1736 (1985).

[42] R. H. McNutt, S. K. Frape, P. Fritz, M. G. Jones, I. M. MacDonald, The ^87^Sr^86^Sr values of Canadian Shield brines and fracture minerals with applications to groundwater mixing, fracture history, and geochronology. Geochim. Cosmochim. Acta 54, 205–215 (1990).

[43] D. Bottomley, A review of theories on the origins of saline waters and brines in the Canadian Precambrian Shield. *Tech. Rep. 17*, Atomic Energy Control Board, Ottawa, Canada (1996).

[44] P. Méjean, D. L. Pinti, M. Larocque, B. Ghaleb, G. Meyzonnat, S. Gagné, Processes controlling ^234^U and ^238^U isotope fractionation and helium in the groundwater of the St. Lawrence Lowlands, Quebec: The potential role of natural rock fracturing. Appl. Geochem. 66, 198–209 (2016).

[45] R. M. Dunk, R. A. Mills, W. J. Jenkins, A reevaluation of the oceanic uranium budget for the Holocene. Chem. Geol. 190, 45–67 (2002).

[46] M. Gascoyne, High levels of uranium and radium in groundwaters at Canada’s Underground Research Laboratory, Lac du Bonnet, Manitoba, Canada. Appl. Geochem. 4, 577–591 (1989).

[47] C. J. Batchelor, I. J. Orland, S. A. Marcott, R. Slaughter, R. L. Edwards, P. Zhang, X. Li, H. Cheng, Distinct permafrost conditions across the last two glacial periods in midlatitude North America. Geophys. Res. Lett. 46, 13318–13326 (2019).

[48] J. B. Murton, R. Peterson, J.-C. Ozouf, Bedrock fracture by ice segregation in cold regions. Science 314, 1127–1129 (2006). 17110573 10.1126/science.1132127

[49] P. Méjean, D. L. Pinti, B. Ghaleb, M. Larocque, Fracturing-induced release of radiogenic ^4^He and ^234^U into groundwater during the last deglaciation: An alternative source to crustal helium fluxes in periglacial aquifers. Water Resour. Res. 53, 5677–5689 (2017).

[50] T. F. Kraemer, T. P. Brabets, Uranium isotopes (^234^U/^238^U) in rivers of the Yukon Basin (Alaska and Canada) as an aid in identifying water sources, with implications for monitoring hydrologic change in arctic regions. Hydrgeol. J. 20, 469–481 (2012).

[51] S. Frape, P. Fritz, R. McNutt, Water-rock interaction and chemistry of groundwaters from the Canadian Shield. Geochim. Cosmochim. Acta 48, 1617–1627 (1984).

[52] S. K. Frape, P. Fritz, The chemistry and isotopic composition of saline groundwaters from the Sudbury Basin, Ontario. Can. J. Earth Sci. 19, 645–661 (1982).

[53] G. S. Boulton, T. Slot, K. Blessing, P. Glasbergen, T. Leijnse, K. van Gijssel, Deep circulation of groundwater in overpressured subglacial aquifers and its geological consequences. Quat. Sci. Rev. 12, 739–745 (1993).

[54] I. D. Clark, M. Douglas, K. Raven, D. Bottomley, Recharge and preservation of Laurentide glacial melt water in the Canadian shield. Ground Water 38, 735–742 (2000).

[55] R. L. Stotler, S. K. Frape, T. Ruskeeniemi, P. Pitkänen, D. W. Blowes, The interglacial-glacial cycle and geochemical evolution of Canadian and Fennoscandian Shield groundwaters. Geochim. Cosmochim. Acta 76, 45–67 (2012).

[56] J.-M. Lemieux, E. A. Sudicky, Simulation of groundwater age evolution during the Wisconsinian glaciation over the Canadian landscape. Environ. Fluid Mech. 10, 91–102 (2010).

[57] J. M. Lemieux, E. A. Sudicky, W. R. Peltier, L. Tarasov, Dynamics of groundwater recharge and seepage over the Canadian landscape during the Wisconsinian glaciation. J. Geophys. Res. Earth 113, F01011 (2008).

[58] S.-E. Lauritzen, J. E. Mylroie, Results of a speleothem U/Th dating reconnaissance from the Helderberg Plateau, New York. J. Caves Karst Stud. 62, 20–26 (2000).

[59] D. Lacelle, B. Lauriol, G. Zazula, B. Ghaleb, N. Utting, I. D. Clark, Timing of advance and basal condition of the Laurentide Ice Sheet during the last glacial maximum in the Richardson Mountains, NWT. Quat. Res. 80, 274–283 (2013).

[60] N. Biller-Celander, J. D. Shakun, D. McGee, C. I. Wong, A. V. Reyes, B. Hardt, I. Tal, D. C. Ford, B. Lauriol, Increasing Pleistocene permafrost persistence and carbon cycle conundrums inferred from Canadian speleothems. Sci. Adv. 7, eabe5799 (2021). 33910910 10.1126/sciadv.abe5799 PMC8081356

[61] S. A. Ewing, J. B. Paces, J. A. O’Donnell, M. T. Jorgenson, M. Z. Kanevskiy, G. R. Aiken, Y. Shur, J. W. Harden, R. Striegl, Uranium isotopes and dissolved organic carbon in loess permafrost: Modeling the age of ancient ice. Geochim. Cosmochim. Acta 152, 143–165 (2015).

[62] T. Blackburn, G. H. Edwards, S. Tulaczyk, M. Scudder, G. Piccione, B. Hallet, N. McLean, J. C. Zachos, B. Cheney, J. T. Babbe, Ice retreat in Wilkes Basin of East Antarctica during a warm interglacial. Nature 583, 554–559 (2020). 32699394 10.1038/s41586-020-2484-5

[63] J. H. Chen, R. L. Edwards, G. J. Wasserburg, ^238^U, ^234^U and ^232^Th in seawater. Earth Planet. Sci. Lett. 80, 241–251 (1986).

[64] E. W. Wolff, H. Fischer, R. Röthlisberger, Glacial terminations as southern warmings without northern control. Nat. Geosci. 2, 206–209 (2009).

[65] U. Hoff, T. L. Rasmussen, R. Stein, M. M. Ezat, K. Fahl, Sea ice and millennial-scale climate variability in the Nordic seas 90 kyr ago to present. Nat. Commun. 7, 12247 (2016). 27456826 10.1038/ncomms12247 PMC4963477

[66] Q. Simon, C. Hillaire-Marcel, G. St-Onge, J. T. Andrews, North-eastern Laurentide, western Greenland and southern Innuitian ice stream dynamics during the last glacial cycle. J. Quat. Sci. 29, 14–26 (2014).

[67] J. T. Andrews, M. E. Kirby, A. Aksu, D. C. Barber, D. Meese, Late Quaternary detrital carbonate (DC-) layers in Baffin Bay marine sediments (67°–74°N): Correlation with Heinrich events in the North Atlantic? Quat. Sci. Rev. 17, 1125–1137 (1998).

[68] A. Gilbert, G. E. Flowers, G. H. Miller, K. A. Refsnider, N. E. Young, V. Radić, The projected demise of Barnes Ice Cap: Evidence of an unusually warm 21st century Arctic. Geophys. Res. Lett. 44, 2810–2816 (2017).

[69] E. P. Verplanck, G. L. Farmer, J. Andrews, G. Dunhill, C. Millo, Provenance of Quaternary glacial and glacimarine sediments along the southeast Greenland margin. Earth Planet. Sci. Lett. 286, 52–62 (2009).

[70] M. H. Walczak, A. C. Mix, E. A. Cowan, S. Fallon, L. K. Fifield, J. R. Alder, J. Du, B. Haley, T. Hobern, J. Padman, S. K. Praetorius, A. Schmittner, J. S. Stoner, S. D. Zellers, Phasing of millennial-scale climate variability in the Pacific and Atlantic Oceans. Science 370, 716–720 (2020). 33004677 10.1126/science.aba7096

[71] Z. D. Sharp, *Principles of Stable Isotope Geochemistry* (University of New Mexico, ed. 2, 2017); 10.25844/h9q1-0p82.

[72] J. Ma, G. Wei, Y. Liu, Z. Ren, Y. Xu, Y. Yang, Precise measurement of stable (δ^88/86^Sr) and radiogenic (^87^Sr/^86^Sr) strontium isotope ratios in geological standard reference materials using MC-ICP-MS. Chin. Sci. Bull. 58, 3111–3118 (2013).

[73] C. L. Batchelor, M. Margold, M. Krapp, D. K. Murton, A. S. Dalton, P. L. Gibbard, C. R. Stokes, J. B. Murton, A. Manica, The configuration of Northern Hemisphere ice sheets through the Quaternary. Nat. Commun. 10, 3713 (2019). 31420542 10.1038/s41467-019-11601-2 PMC6697730

[74] H. Cheng, R. L. Edwards, C. C. Shen, V. J. Polyak, Y. Asmerom, J. Woodhead, J. Hellstrom, Y. Wang, X. Kong, C. Spötl, X. Wang, E. C. Alexander Jr., Improvements in ^230^Th dating, ^230^Th and ^234^U half-life values, and U-Th isotopic measurements by multi-collector inductively coupled plasma mass spectrometry. Earth Planet. Sci. Lett. 371-372, 82–91 (2013).

[75] S. O. Rasmussen, M. Bigler, S. P. Blockley, T. Blunier, S. L. Buchardt, H. B. Clausen, I. Cvijanovic, D. Dahl-Jensen, S. J. Johnsen, H. Fischer, V. Gkinis, M. Guillevic, W. Z. Hoek, J. J. Lowe, J. B. Pedro, T. Popp, I. K. Seierstad, J. P. Steffensen, A. M. Svensson, P. Vallelonga, B. M. Vinther, M. J. C. Walker, J. J. Wheatley, M. Winstrup, A stratigraphic framework for abrupt climatic changes during the Last Glacial period based on three synchronized Greenland ice-core records: Refining and extending the INTIMATE event stratigraphy. Quat. Sci. Rev. 106, 14–28 (2014).

[76] P. Vermeesch, On the visualisation of detrital age distributions. Chem. Geol. 312–313, 190–194 (2012).

[77] D. L. Phillips, P. L. Koch, Incorporating concentration dependence in stable isotope mixing models. Oecologia 130, 114–125 (2002). 28547016 10.1007/s004420100786

[78] P. Vermeesch, IsoplotR: A free and open toolbox for geochronology. Geosci. Front. 9, 1479–1493 (2018).

[79] D. Docquier, L. Perichon, F. Pattyn, Representing grounding line dynamics in numerical ice sheet models: Recent advances and outlook. Surv. Geophys. 32, 417–435 (2011).

[80] C. F. Brædstrup, D. L. Egholm, S. V. Ugelvig, V. K. Pedersen, Basal shear stress under alpine glaciers: Insights from experiments using the iSOSIA and Elmer/Ice models. Earth Surf. Dyn. 4, 159–174 (2016).

[81] S. Tulaczyk, W. B. Kamb, H. F. Engelhardt, Basal mechanics of Ice Stream B, West Antarctica. 2. Undrained-plastic-bed model. J. Geophys. Res. 105, 483–494 (2000).

[82] R. L. Hooke, E. C. Alexander Jr., R. J. Gustafson, Temperature profiles in the Barnes Ice Cap, Baffin Island, Canada, and heat flux from the subglacial terrane. Can. J. Earth Sci. 17, 1174–1188 (1980).

[83] A. Gilbert, G. E. Flowers, G. H. Miller, B. T. Rabus, W. Van Wychen, A. S. Gardner, L. Copland, Sensitivity of Barnes Ice Cap, Baffin Island, Canada, to climate state and internal dynamics. J. Geophys. Res. Earth 121, 1516–1539 (2016).

[84] C. B. Begeman, S. M. Tulaczyk, A. T. Fisher, Spatially variable geothermal heat flux in West Antarctica: Evidence and implications. Geophys. Res. Lett. 44, 9823–9832 (2017).

[85] Y.-C. Yen, Review of thermal properties of snow, ice and sea ice, *Tech. Rep. 81–10*, U.S. Army Cold Regions Research and Engineering Laboratory, Hanover, New Hampshire, USA (1981).

[86] V. Masson-Delmotte, G. Dreyfus, P. Braconnot, S. Johnsen, J. Jouzel, M. Kageyama, A. Landais, M.-F. Loutre, J. Nouet, F. Parrenin, D. Raynaud, B. Stenni, E. Tuenter, Past temperature reconstructions from deep ice cores: Relevance for future climate change. Clim. Past 2, 145–165 (2006).

[87] E. J. Steig, How well can we parameterize past accumulation rates in polar ice sheets? Ann. Glaciol. 25, 418–422 (1997).

[88] J. P. Briner, J. C. Gosse, P. R. Bierman, Applications of cosmogenic nuclides to Laurentide Ice Sheet history and dynamics, in *In Situ-Produced Cosmogenic Nuclides and Quantification of Geological Processes* (Geological Society of America, 2006), vol. 415, pp. 29–41.

[89] J. L. Bamber, R. L. Layberry, S. P. Gogineni, A new ice thickness and bed data set for the Greenland ice sheet: 1. Measurement, data reduction, and errors. J. Geophys. Res. Atmos. 106, 33773–33780 (2001).

[90] B. Hallet, Deposits formed by subglacial precipitation of CaCO_3_. GSA Bulletin 87, 1003–1015 (1976).

[91] G. H. Miller, A. P. Wolfe, E. J. Steig, P. E. Sauer, M. R. Kaplan, J. P. Briner, The Goldilocks dilemma: Big ice, little ice, or “just-right” ice in the Eastern Canadian Arctic. Quat. Sci. Rev. 21, 33–48 (2002).

[92] J. F. Nye, The response of glaciers and ice-sheets to seasonal and climatic changes. Proc. R. Soc. Lond. A 256, 559–584 (1960).

[93] M. R. Koutnik, E. D. Waddington, Well-posed boundary conditions for limited-domain models of transient ice flow near an ice divide. J. Glaciol. 58, 1008–1020 (2012).

[94] B. Cowan, J. Carter, D. Forbes, T. Bell, Postglacial sea-level lowstand on Cumberland Peninsula, Baffin Island, Nunavut. Can. J. Earth Sci. (2021).

[95] R. N. Hiscott, A. E. Aksu, O. B. Nielsen, Provenance and dispersal patterns, Pliocene-Pleistocene section at site 645, Baffin Bay, in *Proceedings of the Ocean Drilling Program, Scientific Results*, S. K. Stewart, Ed. (Ocean Drilling Program, 1989), vol. 105, pp. 31–52.

[96] L. A. Neymark, J. B. Paces, High-precision isotope analysis of uranium and thorium by TIMS. *AGU Fall Meeting Abstracts* V11E-04 (2006).

[97] B. Hamelin, E. Bard, A. Zindler, R. G. Fairbanks, ^234^U/^238^U mass spectrometry of corals: How accurate is the U-Th age of the last interglacial period? Earth Planet. Sci. Lett. 106, 169–180 (1991).

[98] P. M. Chutcharavan, A. Dutton, A global compilation of U-series-dated fossil coral sea-level indicators for the Last Interglacial period (Marine Isotope Stage 5e). Earth Syst. Sci. Data 13, 3155–3178 (2021).

