# Supplementary material for: Terrestrial evidence for ocean forcing of Heinrich events and subglacial hydrologic connectivity of the Laurentide Ice Sheet
Source: Sci Adv. 2022 Oct 19;8(42):eabp9329. doi: 10.1126/sciadv.abp9329 (PMC9581489; doi:10.1126/sciadv.abp9329)
Supplement: Supplementary file 1 — Supplementary Text Figs. S1 to S8 Tables S1 to S3 References [file sciadv.abp9329_sm.pdf]

Science Advances
AAAS

Supplementary Materials for

Terrestrial evidence for ocean forcing of Heinrich events and subglacial hydrologic connectivity of the Laurentide Ice Sheet

Graham H. Edwards *et al.*

Corresponding author: Graham H. Edwards, graham.h.edwards@dartmouth.edu

Sci. Adv. **8**, eabp9329 (2022)
DOI: 10.1126/sciadv.abp9329

The PDF file includes:

Supplementary Text
Figs. S1 to S8
Tables S1 to S3
Legends for data files S1 to S3
References

Other Supplementary Material for this manuscript includes the following:

Data files S1 to S3

Supplementary Text

Calculating accurate U-Th dates from detritus-rich carbonates

U-Th-Sr isotope data for the six subglacial samples are summarized among Figures S3–S7 and tabulated in Data S1. We report U-Th dates and corresponding $\delta^{234}U_o$ without correction for all fractions with (^{230}Th/^{232}Th)>20 (parentheses denote activity ratios), a canonical threshold above which U-Th dates are generally considered insensitive to inherited ^{230}Th (see justifications below). In cases where (^{230}Th/^{232}Th)<20, we use isochron-based correction methods to account for detrital ^{230}Th and ^{234}U contributions. All tabulated and reported U-Th data and dates incorporate systematic uncertainties stemming from uncertainties in isotope tracer composition and decay constants (*74*), unless stated otherwise. Some calculations and plots incorporate only analytical uncertainties to obtain a more precise internal correction and illustrate internal variability, respectively.

We plot projections of three-dimensional U-Th isochrons normalized to ^{238}U (Osmond type diagram) to illustrate the scatter of data. In scenarios where we individually evaluate detrital (^{234}U/^{232}Th) and (^{230}Th/^{232}Th) compositions, we use two-dimensional projections normalized to ^{232}Th (Rosholt type diagram) to aid visualization of detrital compositions at the intercept. All regressions and isochron calculations were performed with IsoplotR (*78*). In cases where the isotopic data are sufficiently overdispersed relative to analytical uncertainties (we choose a threshold of MSWD>5), we use an overdispersion model (*78*) that attributes all overdispersion to natural variability in one of the regressed variables. In the case of two-dimensional Rosholt-type regressions, this overdispersion is attributed to either (^{230}Th/^{232}Th) or (^{234}U/^{232}Th), reflecting heterogeneity in detrital U-Th compositions or calcite-forming water δ^{234}U. In the case of three-dimensional isochron regressions, all overdispersion is attributed entirely to heterogeneity in (^{234}U/^{238}U). Since (^{234}U/^{238}U) consistently shows far greater dispersion relative to analytical

uncertainties than (^{230}Th/^{238}U) when plotted against (^{232}Th/^{238}U) this approach provides a more accurate and precise calculation of U-Th dates than attributing overdispersion to all three isotope ratios.

Samples M09-B177R and M09-B183R both array U-Th isochrons (Fig. S3), indicating that both samples formed in single episodes of calcite precipitation, consistent with their massive textures (Fig. S1). The other four samples, however, do not array straightforward U-Th isochrons, such that (^{234}U/^{238}U) is particularly heterogenous and poorly correlated with (^{232}Th/^{238}U). Since the isochron method assumes a simple two endmember mixture between a ^{232}Th-free authigenic endmember and a thoraniferous detrital endmember of homogenous U-Th isotope composition, isochrons may be perturbed by variability in both the isotopic composition of the detrital component and the δ^{234}U composition of the calcite-forming waters. In the following discussions, we consider and account for these scenarios to calculate U-Th dates of the subglacial precipitate samples.

M09-B184R exhibits a correlation between (^{230}Th/^{238}U) and (^{232}Th/^{238}U), excluding a single high-^{87}Sr/^{86}Sr outlier, but no coherent correlation in the (^{234}U/^{238}U)-(^{232}Th/^{238}U) projection (Fig. S4). This implies a simple two endmember mixing model with respect to (^{230}Th/^{238}U), but substantial heterogeneity in (^{234}U/^{238}U) independent of ^{232}Th content that likely reflects (^{234}U/^{238}U) heterogeneity in the calcite-forming waters. Instead of attempting to constrain a single authigenic ^{230}Th-^{234}U-^{238}U composition, we individually assess the detrital endmember compositions of (^{230}Th/^{232}Th) and (^{234}U/^{232}Th). Figure S4B regresses the ^{238}U-free detrital endmember compositions, identifying a precise detrital (^{230}Th/^{232}Th) of 0.938$\pm$0.132 and (^{234}U/^{232}Th) within uncertainty of zero (at 95 % confidence intervals). We thus interpret this to reflect a significant detrital ^{230}Th component but a negligible ^{234}U contribution. The (^{230}Th/^{232}Th)-dispersion ($0.059^{+0.080}_{-0.030}$) is minor relative to the calculated intercept, and we interpret it as a combination of minor detrital (^{230}Th/^{232}Th) heterogeneity and slight variations in

radiogenic ^{230}Th production due to variable initial (^{234}U/^{238}U) in the different fractions. Since this latter contribution reflects a radiogenic component, we do not include the overdispersion in the uncertainty of our model detrital (^{230}Th/^{232}Th). By subtracting the calculated detrital (^{230}Th/^{232}Th) from the measured compositions, we recalculate Th-corrected U-Th dates of the five low-^{87}Sr/^{86}Sr M09-B184R fractions, which yield concordant ages (Table S1).

We interpret M09-B152R subsamples 1 and 2 (Fig. S1A) separately since they exhibit different ^{87}Sr/^{86}Sr compositions and U-Th system behavior (Fig. S5). M09-B152R.2 arrays an isochron, which we regress with an overdispersion model to account for modest excess dispersion in (^{234}U/^{238}U). In contrast, the (^{234}U/^{238}U)-(^{232}Th/^{238}U) topology of M09-B152R.1 implies a steep correlation, reminiscent of the erratic U-Th behavior observed in M09-B184R. In Figure S5B, we show that the two fractions of M09-B152R.1 regress a detrital (^{230}Th/^{232}Th) within uncertainty of zero, indicating that a detrital Th-correction is not necessary for this modestly radiogenic sample with (^{230}Th/^{232}Th)>8. As in the case of M09-B184R, we attribute the (^{234}U/^{238}U) compositions of M09-B152R.1 to source water heterogeneity rather than the alternative interpretation that it formed from dramatically lower-(^{234}U/^{238}U) waters than its neighboring M09-B152R.2 counterpart. Therefore, we do not calculate or assume a (^{234}U/^{232}Th)-correction and instead accept the uncorrected U-Th dates of M09-B152R.1: 21.78 $\pm$ 0.45 and 22.34 $\pm$ 0.46 ka (relative to 1950 CE). Since the M09-B152R.2 isochron date and uncorrected dates of M09-B152R.1 overlap, we consider these a single calcite-forming event. However, given the complex behavior of M09-B152R.1, we assign the isochron date for M09-B152R.1 as the M09-B152R formation age.

M09-B176R has two well-defined layers with distinct ^{87}Sr/^{86}Sr and U-Th isotope compositions: upper layer M09-B176R.a (subsampled at 5 intervals from a1 at the upper surface to a5 at the base) and lower layer M09-B176R.b (Fig. S6). The less coherent material between layers a and b (M09-B176R.ab) exhibits ^{87}Sr/^{86}Sr and U-Th compositions intermediate between

M09-B176R.a5 and M09-B176R.b. The intermediate compositions and unconsolidated textures indicate that M09-B176R.ab is a mixture of the overlying and underlying components and represents a disconformity at the a-b contact, rather than a discreet calcite-forming event. Thus, we do not report a date for this layer. The lower layer (b) arrays a U-Th isochron that imparts a very minor ^{230}Th-^{234}U-correction, despite the uniformly (^{230}Th/^{232}Th)<20 compositions, and yields an isochron date within uncertainty of the uncorrected U-Th dates of the constituent fractions (Fig. S6).

The upper layer (a) of M09-B176R contains the most radiogenic material in this study. At least one fraction from each subsampled layer exhibits (^{230}Th/^{232}Th)>20, a canonical threshold above which U-Th dates are considered insensitive to detrital U-Th contributions. We report these (^{230}Th/^{232}Th)>20 U-Th dates without correction. In the case of M09-B176R.a, this approach is validated by the following observations. The near-horizontal slope of the (^{230}Th/^{232}Th)-(^{232}Th/^{238}U) projection of the M09-B176R.b isochron (Fig. S6A) indicates that the (^{230}Th/^{232}Th) of this closely related, underlying layer is trivially small with respect to analytical uncertainties. Similarly, the uncorrected U-Th dates of fractions from layers a1 and a4 respectively overlap at 1σ (standard error of the mean) across a range of 17<(^{230}Th/^{232}Th)<20 (Data S1), confirming that these uncorrected U-Th dates do not depend significantly on (^{230}Th/^{232}Th). If we disregard this independence and instead assume that the U-Th systematics of layers a5–a1 record significant detrital Th contamination, we may recalculate U-Th dates corrected for an assumed range of detrital compositions. We recalculate these dates by subtracting (^{230}Th/^{232}Th) compositions drawn from a uniform distribution spanning 0$\leq$(^{230}Th/^{232}Th)$\leq$1 (the highest calculated detrital (^{230}Th/^{232}Th) composition observed in this study is 0.938$\pm$0.132; Fig. S4). The resultant "corrected" mean dates are $\leq$650 years younger but within 2σ (standard error) of their uncorrected counterparts. Thus, for measurements with (^{230}Th/^{232}Th)>20, detrital Th-corrections are both inadvisable based on observed U-Th systematics and do not significantly

alter dates with respect to the interpretations herein.

Overall, the U-Th dates young from the base of the layer (a5) to the top (a1; Fig. S6C), evidencing ongoing carbonate precipitation at the M09-B176R site between 25 and 23 ka. The exception is layer a2, which has older U-Th dates than the underlying a3 layer, and differs texturally from surrounding layers by its coarser detritus and clasts of dark gray material similar to the matrix of layers a1 and a3–a5 (Fig. S6B,C). We conclude that a2 formed during a relatively erosive period that incorporated coarse detritus and rip-up clasts of older (a4, a5) material, resulting in the anomalously old U-Th dates, and we exclude a2 as a calcite precipitation event.

M09-B071R has mm-scale laminations that alternate between dark and light brown as a function of coarse detrital content (Fig. S1F). We sampled these laminations at 8 intervals (Fig. S7). The uppermost interval M09-B071R.a1 has the lowest $^{87}Sr/^{86}Sr$ and the highest ($^{232}Th/^{238}U$) but arrays a U-Th isochron (Fig. S7A). In contrast, layers a2–a8 exhibit a relatively narrow range of $^{87}Sr/^{86}Sr$ and relatively uraniferous compositions of ($^{232}Th/^{238}U$)$<$0.04, comparable to the most radiogenic fractions of M09-B176R (Figs. S6,S7). When regressed as a group, layers a2–a8 yield detrital compositions of ($^{234}U/^{232}Th$) and ($^{230}Th/^{232}Th$) within uncertainty of zero (Fig. S7B), albeit with some scatter. While M09-B071R.a2–8 fractions all exhibit ($^{230}Th/^{232}Th$)$<$20, there is no correlation between the uncorrected U-Th date and ($^{230}Th/^{232}Th$), indicating a minor detrital ^{230}Th component that has negligible leverage on U-Th dates (Fig. S7). Therefore, these uncorrected U-Th dates are accurate estimations of the age of carbonate formation. When compared to their stratigraphic position, these dates span a ca. 3 ka range and are not consistently correlated with stratigraphy. For example, the interior layers a3 and a5 exhibit the youngest dates. The relationship between U-Th dates and stratigraphy imply a complex history of minor open-system behavior and/or post-depositional calcite formation in interior layers a3–a5. Indeed the apparently younger interior ages may reflect continued fluid penetration into the pore-space of uncemented layers with more abundant coarse detrital grains.

Given the approximately normal distribution of dates centered about 18–19 ka (Fig. S7D), we conclude that M09-B071R likely underwent a protracted period of calcite formation that post-dated detrital sediment deposition with minor perturbations by open-system behavior, detrital contamination, and/or incorporation of detrital carbonate (*39*).

In total, we identify 10 distinct Baffin Island subglacial calcite-forming events, summarized in Table S2 along with the corresponding calculated δ^{234}U of the formative waters at the time of calcite precipitation ($\delta^{234}U_o$) and relevant statistics (see also Table 1). Isochron-calculated U-Th dates directly record the age of these calcite formation events, as do the U-Th dates of M09-B176R layers with only one (^{230}Th/^{232}Th)>20 measurement. Therefore, these are reported without alteration. For events with multiple U-Th dates for a single calcite-forming event (M09-B071R, M09-B184R, M09-B176R.a5), we calculate a single weighted mean date and $\delta^{234}U_o$ for the event (using a weighted mean with random effects model algorithm).

Previously published U-Th dates

Refsnider et al. (*22*) reported U-Th dates for four different subglacial precipitates from the BIC and Rimrock Hills localities. They report one isochron-derived U-Th date from the BIC margin of 21.0 $\pm$ 5.4 ka, which overlaps within its uncertainty both the H1 and H2 calcite-forming episodes observed in our data (M09-B153R, Table 1). Since detrital U and Th are accounted for in the isochron-calculated date, we include this date in our interpretations, though its low precision provides little resolution to the subglacial chronologic record. They also report a single-fraction date of 19.1 $\pm$ 0.3 ka for a precipitate from Rimrock Hills, which overlaps the H1 timescale and precedes the mean date of M09-B071R by <1 ka (M09-B064R, Table 1, Fig. 4). The authors report this date without correction on account of its high authigenic U content and low detrital Th content, reflected in a measured (^{230}Th/^{232}Th)>30 and (^{232}Th/^{238}U)<0.01 (*22*). Given the patterns observed in our own data (see above discussions), we consider this radiogenic

U-Th date to be sufficiently rigorous and include it in our interpretations. A third date was reported for two different precipitate rocks (M09-B190R, M09-B181R) based on an assumed two-component mixing model between a single fraction measurement from each sample (*22*). In the absence of *a priori* evidence for a genetic relationship between these samples and without sufficient data to corroborate the linear mixing model (requires n >2 fractions), the assumed mixing relationship is not rigorous, and we exclude this date from all interpretations of the present study.

Simplified quantitative model of basal melting & freezing

We conjecture that the carbonate subglacial precipitate samples formed during Heinrich events (HE) because basal thermal conditions in the sample collection region switched from basal freezing to basal melting during these events. Our conceptual model links these transitions in basal thermal regime to increased ocean thermal forcing during HEs (*10, 13*), which set off grounding line retreat of the outlet glaciers that drained the eastern flank of the Foxe Dome into Baffin Bay (e.g. through Buchan Gulf and Scott Inlet). Grounding line retreat triggered ice flow acceleration and increases in gravitational driving stress, the latter due to steepening of ice surface slopes (*79*). This results in enhanced basal shear heating, which is a product of ice velocity and basal shear stress (*26*). In the rocky plateau setting of interior Baffin Island, we assume the basal shear stress to be equal in magnitude to the gravitational driving stress, i.e. a zeroth-order shallow ice approximation (*80*).

Here we demonstrate the plausibility of our conceptual model by examining a simplified, zeroth-order model of basal thermal energy balance between and during HEs. Our simple analysis is analogous to the theoretical basis for the binge/purge model of HEs proposed by MacAyeal (*8*). We start with the simplest formulation for the basal thermal energy balance, E, beneath a polar ice sheet with two sources of heat — the geothermal flux, G, and shear heating,

S — and one sink of heat — the conductive heat loss, Q (*81*):

$$E = G + S - Q \tag{1}$$

The geothermal flux, G, represents a time-independent boundary condition on this problem. Prior research on Barnes Ice Cap indicates that the geothermal flux in this region is very low, $\sim$30 mW/m^2, as compared to typical continental heat fluxes (*82–84*).

To obtain an estimate of the conductive heat loss, Q, we use the analytical solution for a 1D vertical advection-diffusion steady-state ice temperature profile, see equation 1 in (*84*). We assume ice thickness of 1500 m in the region of sample collection (*35*) and thermal conductivity for glacier ice of 2.1 W m^{-1} K^{-1} (*85*). The two climate parameters used in this calculation are the ice surface temperature and accumulation rate. Given the simplified nature of our analysis, we set the basal temperature to 0 °C. The ice surface temperature of modern Barnes Ice Cap is -10 °C (*82*). To account for the higher ice surface elevation and cooler last glacial maximum (LGM) climate (*86*), our calculations use ice surface temperatures of ca. -30 °C, see also (*8*). Similarly, the upper parts of the accumulation zone of the Barnes Ice Cap receive at present $\sim$0.3 m/yr of ice equivalent per year (*83*). Ice core data indicate that the LGM accumulation rates were about half of modern, and LGM ice surface elevations higher than modern elevations imply even less accumulation (*87*). Hence, we take the LGM ice accumulation rate to be 0.1 m/yr, one-third of modern. Based on these assumptions, we calculate a conductive heat loss of $\sim$70 mW/m^2.

In the absence of basal shear heating, the geothermal flux and the conductive heat loss for this representative scenario would result in basal freezing conditions with a negative energy budget of –40 mW/m^2. We approximate the rate of basal heat generation accompanying ice motion as the product of ice velocity and basal shear stress (*26*). Ice velocity is estimated as the balance velocity in the region of sample collection, which is about 500 km east of the

ice divide of the Foxe Dome (*35, 88*). At the above-mentioned accumulation rate of 0.1 m/yr, the total annual surface input of ice along a 500 km–long flowline, expressed per meter of ice sheet width, amounts to 50 000 m^2/yr. We calculate a balance velocity of $\sim$30 m/yr by dividing this annual volume flux per unit width by the estimated LGM ice thickness of 1500 m in the sampling region (*35*). Basal shear stress represents the least constrained parameter in our calculations. Figure S8 shows the linear dependence of the basal heat budget on basal shear stress for the representative case discussed here. The transition between freezing and melting occurs at a reasonable basal shear stress of about 42 kPa (*89*).

A hypothetical state representing basal freezing conditions in the sample collection region between HEs is shown by the solid purple star in Figure S8. During HEs grounding line destabilization triggered ice acceleration which propagated upstream towards the sampling region. Grounding line retreat also results in steepening of ice surface slope and an associated increase in the driving stress and, per the shallow ice approximation, the basal stress. This will result in enhanced shear heating during HEs shown by the open purple star, which assumes a 10 % increase in velocity and a 10 kPa increase in basal stress. The resulting positive heat budget is 10 mW/m^2 and corresponds to approximately 1 mm/yr of basal melting. We note that onset of basal melting may enhance basal sliding, which can be important in the formation of calcite precipitates during ice sliding over bedrock bumps (*90*). Although the representative example shown here is non-unique, we use it to demonstrate that there is a glaciologically plausible link between a climatically driven grounding line retreat, basal melting, and subglacial precipitation of calcite.

Lag time between grounding line retreat and onset of basal melting in the sampling region

One concern that may be raised about our analysis pertains to the speed with which perturbations originating at the grounding line would propagate to the sample collection region $\sim$180 km

away (*88, 91*). Propagation of glaciological perturbations takes place through kinematic and diffusive processes (*92*). Below we argue that the expected time needed to propagate perturbations from the grounding line region to the study region is short compared to the uncertainty in the U-Th dates of subglacial carbonate precipitates reported in this study.

Kinematic waves on glaciers travel at 2–5 times the ice velocity itself (*92*). Above we estimated the balance velocity in the sampling region to be $\sim$30 m/yr. At the grounding line, ice thickness is estimated to be only 500 m, see figure 2 in (*91*). This implies velocity at the grounding line that is at least three times faster than in the study region (based on the difference in ice thickness of 500 m versus 1500 m). However, ice flowing across the eastern flank of Baffin Island did not move as a uniform sheet but had to converge into discrete fjords, whose widths account for approximately one-third to one-fourth of the island's eastern edge (*91*). Combined, these two effects suggest that the ice velocity was about an order of magnitude faster, $\sim$300 m/yr, in the fjords and at the grounding line than upstream (30 m/yr). Kinematic waves induced by grounding line perturbations would thus travel quite quickly through fjords, 600–1500 m/yr, and traverse their lengths ($\sim$130 km, see figure 2 in (*91*)) within about a century. They would travel ten times slower, 60–150 m/yr, up the remaining 50 km of the ice sheet flowline. Altogether, it would take approximately 400–1000 years for the ice sheet overlying the sample collection region to respond to perturbations originating at the grounding line through kinematic waves alone. This estimate can be shortened by almost one-half if we add diffusive processes for propagation of perturbations, which in the ice sheet portion of the flowline should operate about as fast as the kinematic component (*92*). Our best estimate is a reaction time of about 250–650 years, which is comparable to the uncertainties in the U-Th dates of our samples.

Perturbation of Foxe Dome by the Hudson Strait Ice Stream

A potential alternative explanation to the one outlined above is to assume that the subglacial conditions in the sample collection area varied with time because of the lowering of Foxe Dome caused by activation of Hudson Strait Ice Stream (HSIS) during HEs (*91*). However, the changes in subglacial conditions recorded in our samples are essentially simultaneous with HEs, whereas propagation of dynamic signals near and across ice divides is relatively slow (*93*). As mentioned above, kinematic waves propagate at 2–5 times the ice velocity, which becomes nil near an ice divide. Diffusion of perturbations is then the predominant process for glaciological adjustments in the slow-moving portions of ice sheets. However, the formulation for the diffusion coefficient also shows a direct dependence on ice velocity, which is low near ice divides, e.g. equation 45 in (*92*). Hence, propagation of thinning across the ice divide would require timescales that are comparable to the adjustment timescale for an entire large ice sheet (e.g. 5 ka, see p. 576 in (*92*)). The more parsimonious explanation is that changes in subglacial conditions in our study region were forced by variations in grounding line positions along the eastern edge of Baffin Island.

Was Baffin Island ice streaming a secondary response to Heinrich Events?

We substantiate a model that eastern Baffin Island ice streams were destabilized by subsurface ocean warming that concurrently destabilized the HSIS. An alternative hypothesis to explain the concordance is that marine ice stream destabilization on Baffin Island was a secondary response to the ocean-climate consequences of HEs (e.g. sea level rise) rather than a primary response to the HE stimulus. While the record of subglacial melting presented here lacks the temporal resolution to evaluate whether ice stream surging on Baffin Island predates such secondary processes, both sea level reconstructions and the marine sedimentary record of Baffin Bay contradict this hypothesis.

Dated marine limits, submerged coastal features, and glacial isostatic adjustment models of the Cumberland Peninsula of southeast Baffin Island indicate that local Holocene sea levels initially fell in response to deglacial unloading and isostatic rebound (*94*). While we are unaware of any studies examining the isostatic response of Baffin Island to glacial unloading of the HSIS and Foxe Dome during HEs, the postglacial reconstructions imply that relative sea levels in eastern Baffin Bay may have actually fallen during HEs. Such conditions of lower sea level are expected to have a comparatively stabilizing effect on eastern Baffin Island ice streams, rather than the destabilized response recorded by the subglacial precipitate record.

A prominent feature of Baffin Bay marine sediments are carbonate IRD derived from Paleozoic outcrops in northern Baffin Bay, e.g. (*95*). Some of these Baffin Bay detrital carbonate (BBDC) layers were deposited during interstadials and imply enhanced discharge of IRD-laden icebergs from ice streams terminating in northern Baffin Bay (e.g. Lancaster Sound) between HEs (*66, 67*). In a core from eastern Baffin Bay, sediments broadly linked to eastern Baffin Island provenance are also represented in BBDC layers (*66*), suggesting that many marine terminating ice sheets in Baffin Bay responded to the surface ocean-atmosphere conditions of interstadials. However, our results show that only HE conditions were sufficient to elicit subglacial melting over interior Baffin Island, while such conditions were not conducive to iceberg flux from northern Baffin Bay and BBDC deposition. Like interstadials, the ocean-climate responses to HEs are likely to be concentrated at the ocean surface (e.g. sea level rise). Thus, the decoupling of regional subglacial melting on Baffin Island during HEs from changing ocean surface conditions in Baffin Bay during interstadials contradicts the hypothesis that the former might result from ocean surface responses to HEs. Rather, this requires a different stimulus uniquely linked to HEs, for which subsurface ocean warming is most viable, as outlined in the main text.

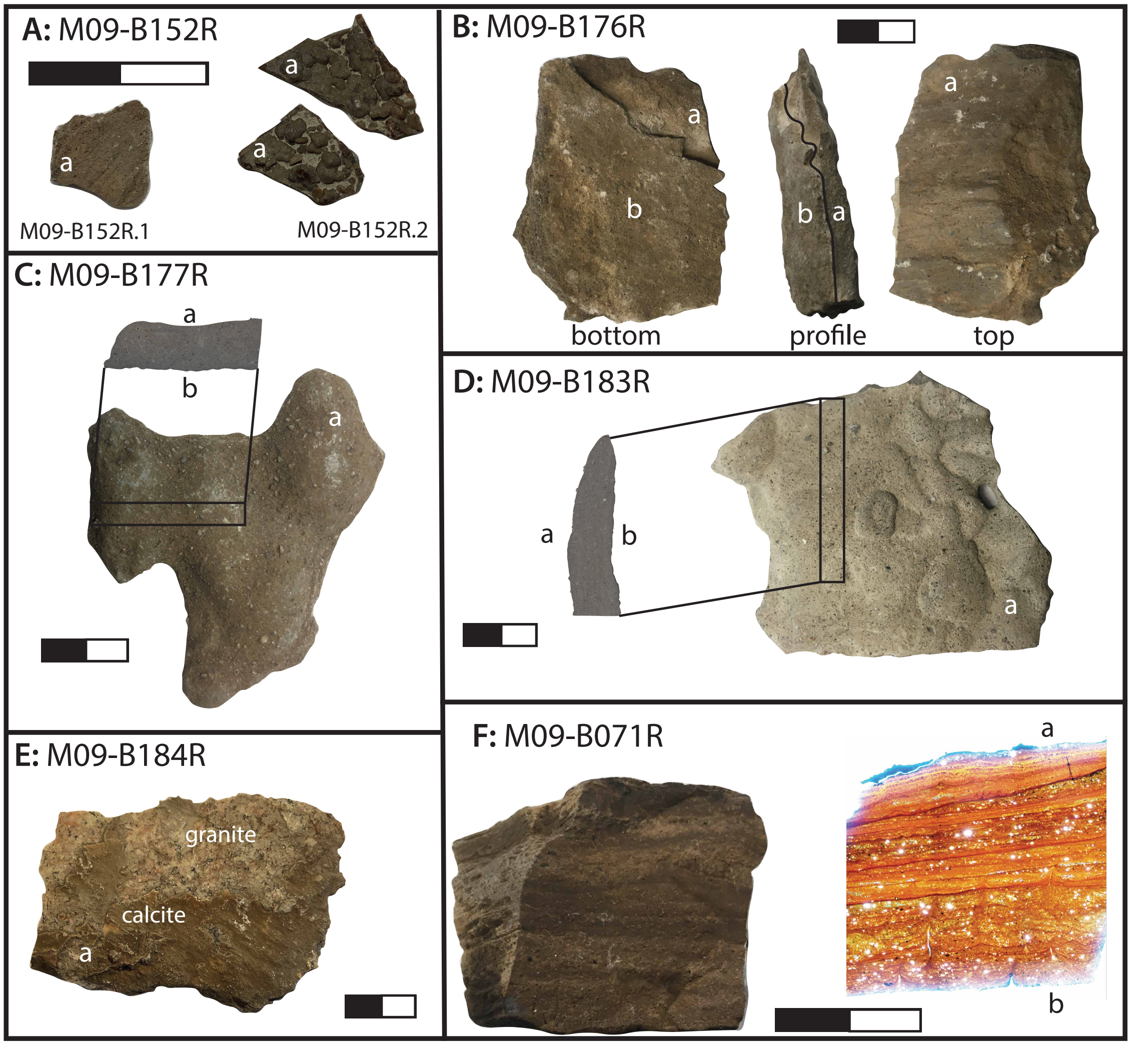

Fig. S1. Subglacial carbonate precipitates analyzed in this study. Scale bars represent 2 cm. Labels "a" and "b" respectively indicate upper and lower surfaces/layers. **A.** M09-B152R fragments are mm-scale veneers from rock surfaces, exhibiting two different lithologies. M09-B152R.1: light-brown carbonate-cemented clastics with linear grooves parallel to the direction of ice flow. M09-B152R.2: dark brown, mm-scale nodules with less detrital content than M09-B152R.1. **B.** M09-B176R has two distinct layers (a and b), separated by a sharp contact (traced in "profile" perspective). See Fig. S6 for cross-section. **C.** M09-B177R is gray-brown with a globular surface morphology and a massive interior texture of calcite-cemented sand-to-silt detritus. Black box outlines location of cross-section. **D.** M09-B183R is pale grey to beige with a globular surface morphology and a massive calcite-cemented interior texture similar to M09-B177R. Cross-section outlined as in C. This rock was sampled within 60 m of the Barnes Ice Cap margin, suggesting very recent exposure, which may account for its lighter surface color compared to the morphologically similar M09-B177R. **E.** M09-B184R is a thin, light brown veneer of calcite on a granite clast, with delicate fluted lineations parallel to local ice flow. **F.** M09-B071R, sampled from the Rimrock Hills region, is a calcite-cemented siltstone with alternating dark and light brown laminations. Light layers correspond to increased detrital sand content (visible in transmitted light photomicrograph).

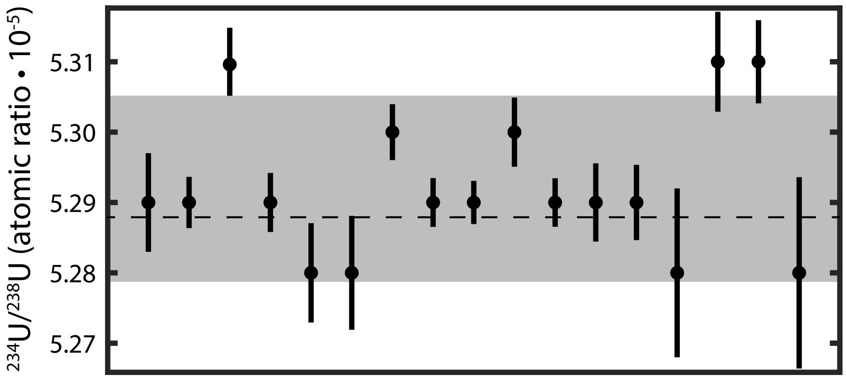

Fig. S2. Measurements of standard reference material (SRM) 4321b collected over the course of this study confirm U isotope reproducibility. Gray bar represents the 1σ (standard deviation, $\sim$0.25 %) envelope about the long-term laboratory mean ($^{234}U/^{238}U = 5.29195 \cdot 10^{-5}$, n=72), which overlaps the reported value of (*96*): $^{234}U/^{238}U = 5.2879 \cdot 10^{-5}$ (dashed line).

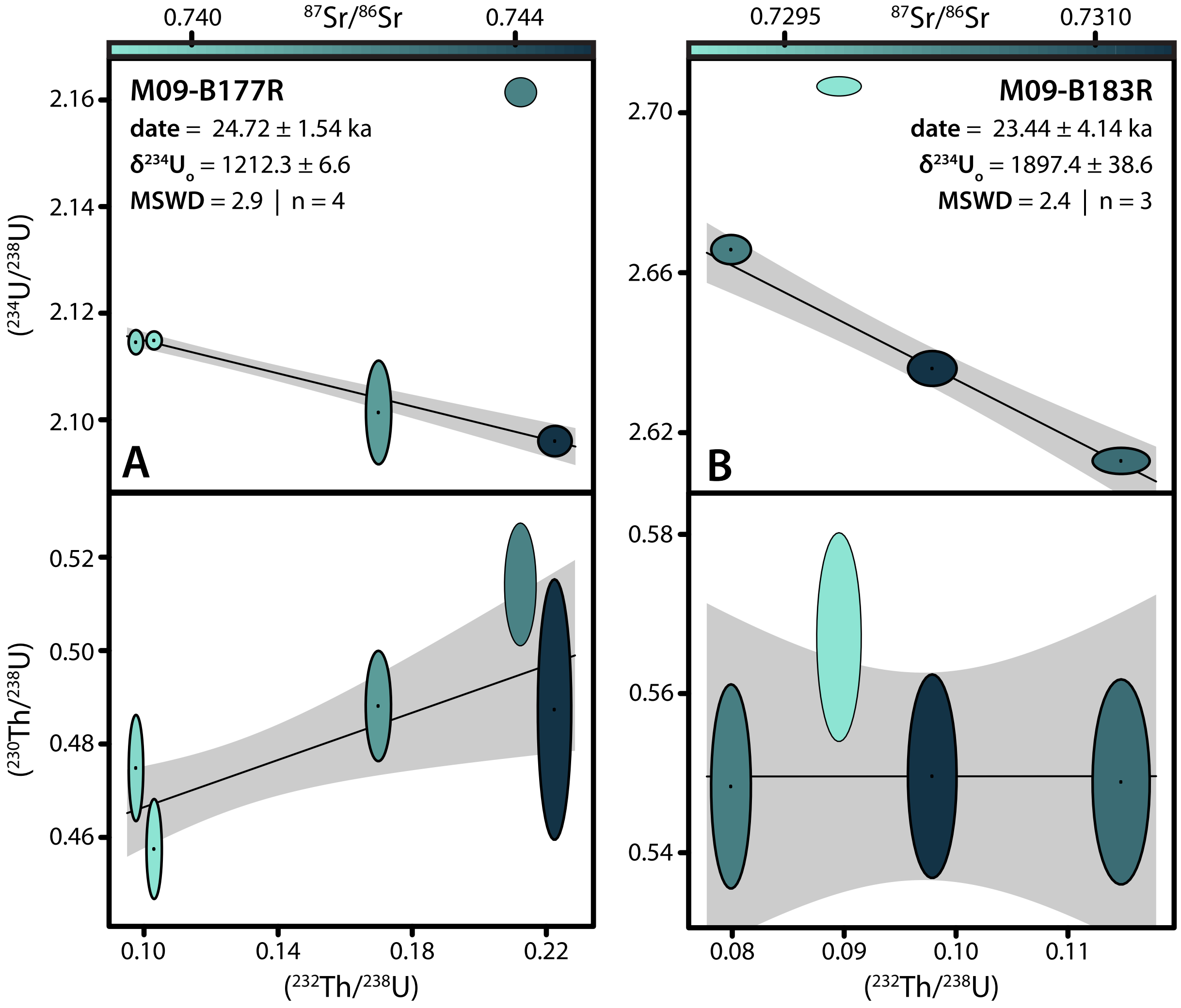

Fig. S3. Two-dimensional projections of three-dimensional U-Th isochrons for M09-B177R (**A**) and M09-B183R (**B**). Color denotes $^{87}Sr/^{86}Sr$. All reported and plotted uncertainties (ellipses and gray regression envelopes) reflect 95 % confidence intervals and include decay constant and tracer uncertainties. Fractions included in the regressions are indicated with black dots and thick borders.

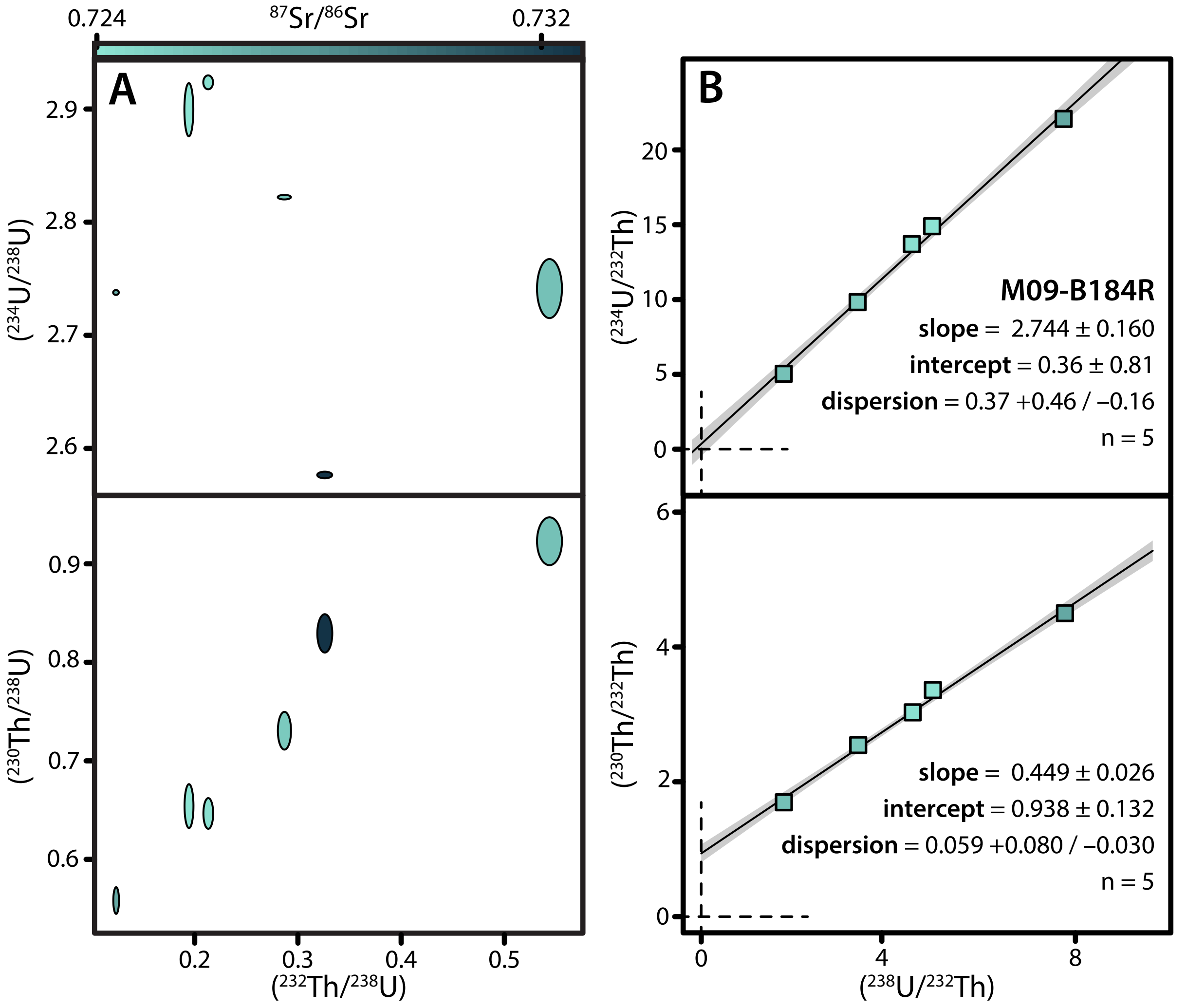

Fig. S4. U-Th isotope and $^{87}Sr/^{86}Sr$ compositions of M09-B184R fractions. **A.** Low $^{87}Sr/^{86}Sr$ fractions are apparently correlated with respect to ($^{230}Th/^{238}U$)-($^{232}Th/^{238}U$) but not ($^{234}U/^{238}U$)-($^{232}Th/^{238}U$). Ellipses reflect 95 % confidence intervals, including decay constant and tracer uncertainties. **B.** Regressions of ^{232}Th-normalized ^{230}Th-^{234}U-^{238}U data identify a significantly non-zero ($^{230}Th/^{232}Th$)-intercept and an ($^{234}U/^{232}Th$)-intercept within uncertainty of zero (dashed lines). Regressions incorporate analytical uncertainties only (smaller than symbols) and were calculated with a maximum likelihood estimation method that attributes over-dispersion to geologic variation in ($^{234}U/^{232}Th$) or ($^{230}Th/^{232}Th$), summarized in the reported "dispersion" terms (*78*). Plotted and reported uncertainties are 95 % confidence intervals.

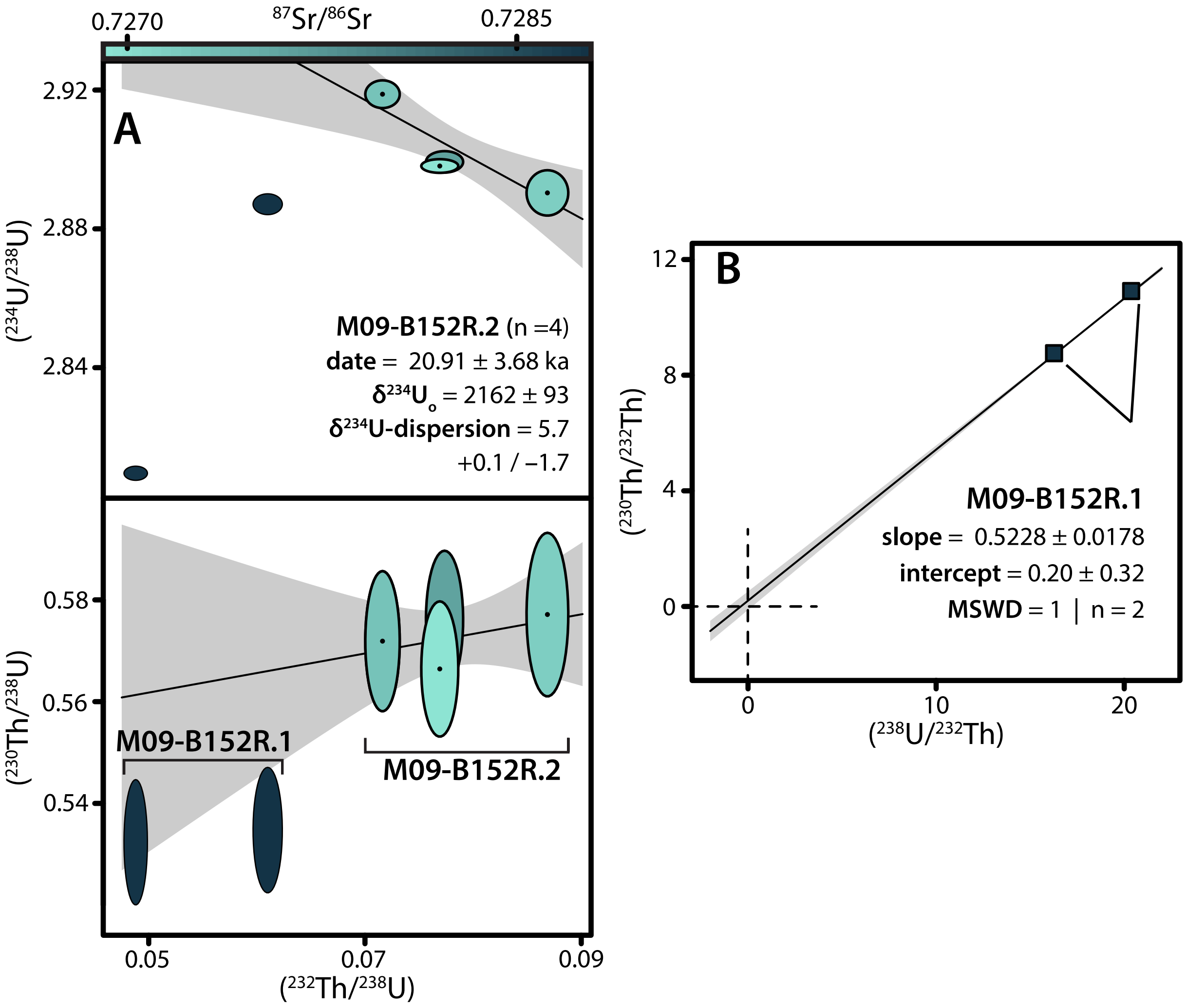

Fig. S5. U-Th isotope and $^{87}Sr/^{86}Sr$ compositions of M09-B152R fractions. **A.** M09-B152R.1 and M09-B152R.2 exhibit distinct Sr isotope compositions and U-Th behavior. All plotted uncertainties (ellipses and gray regression envelopes) and reported uncertainties reflect 95 % confidence intervals and include tracer and decay constant uncertainties. Regression of M09-B152R.2 fractions was calculated by a maximum likelihood estimation method that attributes over-dispersion to geologic variability in ($^{234}U/^{238}U$) (*78*). **B.** Regression of M09-B152R.1 ($^{230}Th/^{232}Th$)-($^{238}U/^{232}Th$) data intersects the origin (dashed lines) within a 95 % confidence interval (gray envelope). Regression incorporates analytical uncertainties only (smaller than symbols).

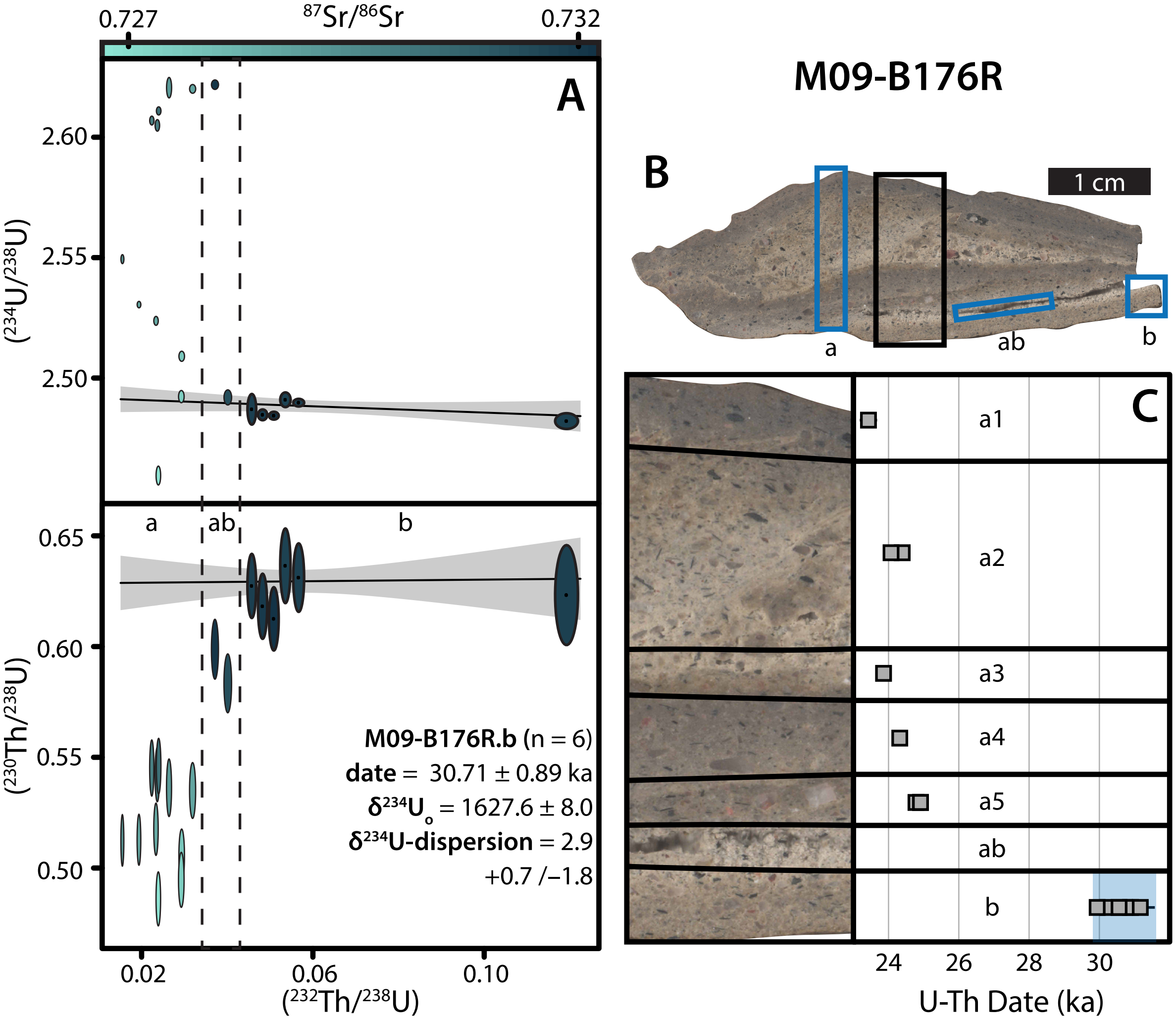

Fig. S6. A. Two-dimensional projections of three-dimensional U-Th isotope plots and $^{87}Sr/^{86}Sr$ compositions of M09-B176R fractions. Subsample groups a, ab, and b (see **B**) are separated by vertical dashed lines. Fractions of subsample M09-B176R.b exhibit similarly elevated $^{87}Sr/^{86}Sr$ compositions and array a U-Th isochron (regressed fractions have black central dots and thick ellipse borders). All reported and plotted uncertainties (ellipses and gray regression envelopes) reflect 95 % confidence intervals and include tracer and decay constant uncertainties. Regression was calculated by a maximum likelihood estimation method that attributes over-dispersion to geologic variability in $(^{234}U/^{238}U)$ (*78*). **B.** Cross-section of M09-B176R. Blue boxes indicate sampling locations of upper unit (a), lower unit (b), and less coherent material between those units (ab). Black box bounds enlarged region in panel C. **C.** Uncorrected U-Th dates of stratigraphic layers of M09-B176R, plotted with analytical uncertainties (2σ standard error, typically smaller than symbols). Black lines demarcate layer divisions. M09-B176R.b U-Th dates are within uncertainty of the isochron date (blue). All plotted M09-B176R.a dates reflect fractions with $(^{230}Th/^{232}Th)>20$.

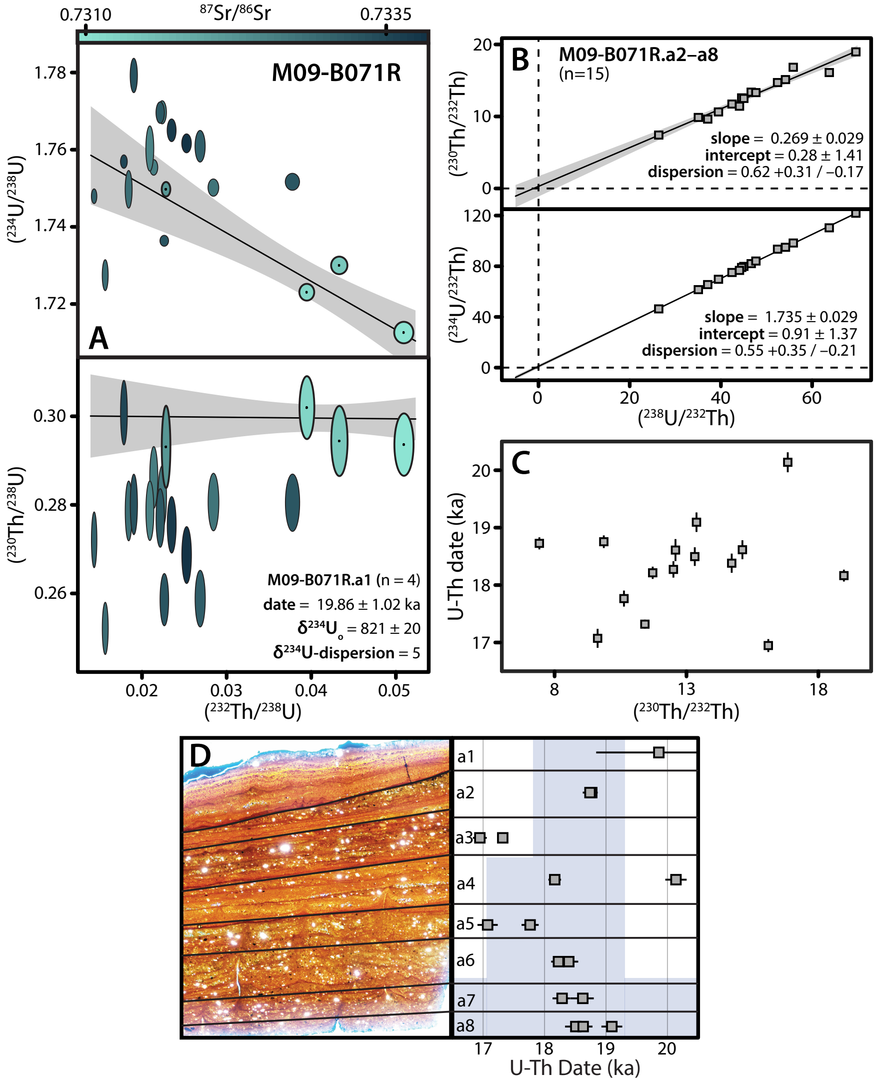

Fig. S7. **A.** Two-dimensional projections of three-dimensional U-Th isotope plots and $^{87}Sr/^{86}Sr$ compositions of M09-B071R fractions. M09-B071R.a1 (see **D**) exhibits the four lowest $^{87}Sr/^{86}Sr$ compositions and arrays a U-Th isochron (regressed fractions identified with black center dots and thick ellipse borders). Remaining fractions show no apparent trend in U-Th isotopes or $^{87}Sr/^{86}Sr$. Reported and plotted uncertainties (ellipses and gray regression envelopes) reflect 95 % confidence intervals that include tracer and decay constant uncertainties. Regression was calculated by a maximum likelihood estimation method that attributes over-dispersion to geologic variability in ($^{234}U/^{238}U$) (*78*). Uncertainty on $\delta^{234}U$ dispersion is smaller than the reported significant figures on δ^{2} $^{34}U_o$. **B.** Regressions of ^{232}Th-normalized ^{230}Th-^{234}U-^{238}U data of the remaining M09-B071R layers (a2–a8) identify detrital ($^{234}U/^{232}Th$) and ($^{230}Th/^{232}Th$) contributions within uncertainty of zero (dashed lines). Regressions incorporate analytical uncertainties only (smaller than symbols) and were calculated with a maximum likelihood estimation method that attributes over-dispersion to geologic variation in ($^{234}U/^{232}Th$) or ($^{230}Th/^{232}Th$) (*78*). Gray envelopes and reported uncertainties are 95 % confidence intervals. **C.** Uncorrected U-Th dates of fractions from layers a2–a8 exhibit no dependency on ($^{230}Th/^{232}Th$) ($\pm 2\sigma$ standard error analytical uncertainties). **D.** U-Th dates relative to stratigraphic position. Layer a1 date from isochron (**A**). Layers a2–a8 are uncorrected single-fraction dates ($\pm 2\sigma$ standard error analytical uncertainties). Black lines demarcate layer divisions. Pale blue bars reflect histogram distribution of mean values (bin width=0.75 ka; panel height=5 counts).

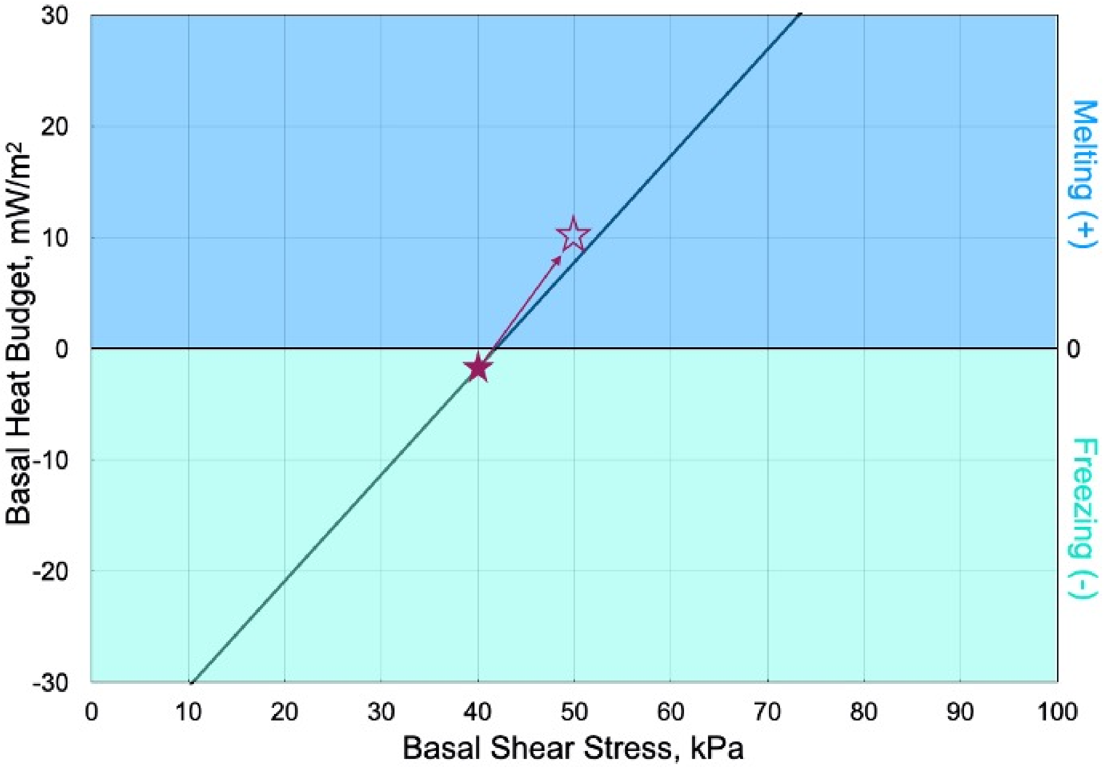

Fig. S8. Simulated basal heat budget at a range of basal shear stress conditions for the representative case considered in the text (dark blue line). Positive heat budget corresponds to basal melting and negative heat budget to basal freezing. The solid purple star represents the hypothetical basal freezing state near the sample collection site (modern Barnes Ice Cap margin). An enhanced shear heating state (open purple star) corresponds to a 10 % increase in ice velocity and 10 kPa increase in basal shear stress. See Supplementary Text for further discussion.

Table S1. Detrital Th-corrected U-Th dates (calculated relative to 1950 CE datum) and initial $\delta^{234}U$ ($\delta^{234}U_o$) for sample M09-B184R, calculated by subtracting the detrital ($^{230}Th/^{232}Th$) composition regressed in Fig. S4. Uncertainties are absolute 2σ standard error and include systematic (i.e. tracer, decay-constant) uncertainties.

Fraction	U-Th date (ka)	$\delta^{234}U_o$ (‰)
M09-B184R-2	17.69 ± 1.29	2022.4 ± 8.9
M09-B184R-3	17.44 ± 3.37	1829.5 ± 27.8
M09-B184R-4	18.92 ± 1.34	2004.0 ± 20.9
M09-B184R-5	18.78 ± 0.87	1832.8 ± 4.9
M09-B184R-6	19.03 ± 1.80	1923.0 ± 9.9

Table S2. Model U-Th ages and initial $\delta^{234}U$ ($\delta^{234}U_o$) compositions of central Baffin Island subglacial calcite-forming events, calculated from isochron regressions, individual fractions, or weighted means. Means are calculated from uncorrected U-Th dates and $\delta^{234}U_o$ of fractions unless otherwise stated. In the case where the MSWD>5, we report the calculated dispersion ("disp.") of the random effects model in lieu of a MSWD. Ages are relative to a 1950 CE datum. All uncertainties reflect 95 % confidence intervals and include systematic (i.e. tracer, decay-constant) uncertainties.

Sample	Method	n	Model age (ka)	MSWD	$\delta^{234}U_o$ (‰)	Dispersion
M09-B071R	mean[1]	16	18.38 ± 0.40	disp. = $0.80^{+0.41}_{-0.24}$	797.5 ± 7.1	$14.3^{+7.1}_{-4.0}$
M09-B184R	mean[2]	5	18.56 ± 0.58	0.78	1923 ± 71	81^{+96}_{-33}
M09-B152R	isochron	4	20.91 ± 3.68	—	2162 ± 93	$5.7^{+0.1}_{-1.7}$
M09-B183R	isochron	3	23.44 ± 4.14	2.4	1897.4 ± 38.6	—
M09-B177R	isochron	4	24.72 ± 1.54	2.9	1212.3 ± 6.6	—
M09-B176R.a1	—	1	23.43 ± 0.53	—	1559.6 ± 4.0	—
M09-B176R.a3	—	1	23.86 ± 0.49	—	1657.6 ± 2.8	—
M09-B176R.a4	—	1	24.32 ± 0.53	—	1736.0 ± 4.4	—
M09-B176R.a5	mean	3	24.87 ± 0.29	0.085	1724.8 ± 3.3	$2.5^{+6.0}_{-2.1}$
M09-B176R.b	isochron	6	30.71 ± 0.89	—	1627.6 ± 8.0	$2.9^{+0.7}_{-1.8}$

[1]Calculated from n=15 individual fraction dates and n=1 isochron date (B071R.a1, Fig. S7)
[2]Calculated from dates in Table S1.

Table S3. Carbonate C and O isotope compositions of Baffin Island subglacial precipitates. Subsample labels follow the labelling schemes of Figs. S1, S6, and S7 for U-Th-Sr subsampling. Uncertainties of $\delta^{13}C$ and $\delta^{18}O$ measurements are <0.05 and <0.10 ‰, respectively.

Sample.Subsample	Weight % $CaCO_3$	$\delta^{13}C_{(VPDB)}$ (‰)	$\delta^{18}O_{(VSMOW)}$ (‰)	$\delta^{18}O_{(VPDB)}$ (‰)
M09-B071R.a1	69.1	-9.40	4.69	-25.43
M09-B071R.a2	66.3	-9.79	5.09	-25.05
M09-B071R.a3	56.5	-9.82	5.26	-24.88
M09-B071R.a4	73.0	-9.59	5.12	-25.02
M09-B071R.a5	65.2	-9.39	4.76	-25.37
M09-B071R.a6	62.6	-9.26	4.41	-25.71
M09-B071R.a7	67.3	-9.22	4.64	-25.48
M09-B071R.a8	65.1	-9.19	4.39	-25.72
M09-B15R2.1	75.2	-7.33	5.99	-24.17
M09-B152R.2	85.1	-7.93	6.11	-24.06
M09-B176R.a1	71.7	-7.97	6.69	-23.49
M09-B176R.a2	52.9	-7.70	6.99	-23.20
M09-B176R.a3	72.4	-7.47	7.28	-22.92
M09-B176R.a4	64.3	-8.07	6.90	-23.29
M09-B176R.a5	57.9	-8.01	6.88	-23.31
M09-B176R.b	59.8	-8.21	6.14	-24.03
M09-B177R.a	53.4	-8.49	7.28	-22.92
M09-B177R.b	33.1	-7.42	8.05	-22.17
M09-B183R.a	47.8	-8.12	7.82	-22.40
M09-B183R.b	46.3	-7.71	6.45	-23.73
M09-B184R	80.0	-7.56	5.69	-24.46

Data S1 (separate file)

Carbonate U-Th and $^{87}Sr/^{86}Sr$ data from Baffin Island subglacial precipitates and Marine Isotope Stage (MIS) 5e coral standard, previously reported as J2-A and J2-B in (*97*) and recalculated with updated decay constants as HA91-005 in (*98*) (dataset Version 1.15), with reported U-Th dates ranging 120.8–135.6 ka and $\delta^{234}U_o$ = 153.2–191.0 ‰. All uncertainties are 2σ absolute standard error. Parentheses indicate activity ratios. All U-Th data include tracer uncertainties. U-Th dates are calculated relative to 1950 CE and include tracer and decay constant uncertainties. Decay constants from (*74*). Subsample naming scheme follows Table S2 and Figs. S1, S6, and S7.

Data S2 (separate file)

Machine-readable format of Table S2.

Data S3 (separate file)

Machine-readable format of Table S3.

REFERENCES AND NOTES

1. A. S. Dyke, J. T. Andrews, P. U. Clark, J. H. England, G. H. Miller, J. Shaw, J. J. Veillette, The Laurentide and Innuitian ice sheets during the Last Glacial Maximum. *Quat. Sci. Rev.* **21**, 9–31 (2002).

2. P. U. Clark, A. S. Dyke, J. D. Shakun, A. E. Carlson, J. Clark, B. Wohlfarth, J. X. Mitrovica, S. W. Hostetler, A. M. McCabe, The Last Glacial Maximum. *Science* **325**, 710–714 (2009).

3. L. C. Menviel, L. C. Skinner, L. Tarasov, P. C. Tzedakis, An ice–climate oscillatory framework for Dansgaard–Oeschger cycles. *Nat. Rev. Earth Environ.* **1**, 677–693 (2020).

4. S. R. Hemming, Heinrich events: Massive late Pleistocene detritus layers of the North Atlantic and their global climate imprint. *Rev. Geophys.* **42**, RG1005 (2004).

5. Y. J. Wang, H. Cheng, R. L. Edwards, Z. S. An, J. Y. Wu, C.-C. Shen, J. A. Dorale, A high-resolution absolute-dated Late Pleistocene monsoon record from Hulu Cave, China. *Science* **294**, 2345–2348 (2001).

6. W. F. Ruddiman, Late Quaternary deposition of ice-rafted sand in the subpolar North Atlantic (lat 40° to 65°N). *GSA Bulletin* **88**, 1813–1827 (1977).

7. H. Heinrich, Origin and consequences of cyclic ice rafting in the Northeast Atlantic Ocean during the past 130,000 years. *Quatern. Res.* **29**, 142–152 (1988).

8. D. R. MacAyeal, Binge/purge oscillations of the Laurentide Ice Sheet as a cause of the North Atlantic's Heinrich events. *Paleoceanography* **8**, 775–784 (1993).

9. R. Zahn, J. Schönfeld, H.-R. Kudrass, M.-H. Park, H. Erlenkeuser, P. Grootes, Thermohaline instability in the North Atlantic during meltwater events: Stable isotope and ice-rafted detritus records from Core SO75-26KL, Portuguese Margin. *Paleoceanogr. Paleoclimatol.* **12**, 696–710 (1997).

10. S. A. Marcott, P. U. Clark, L. Padman, G. P. Klinkhammer, S. R. Springer, Z. Liu, B. L. Otto-Bliesner, A. E. Carlson, A. Ungerer, J. Padman, F. He, J. Cheng, A. Schmittner, Ice-shelf

collapse from subsurface warming as a trigger for Heinrich events. *Proc. Natl. Acad. Sci. U.S.A.* **108**, 13415–13419 (2011).

11. A. E. Jennings, J. T. Andrews, C. Ó Cofaigh, G. St-Onge, S. Belt, P. Cabedo-Sanz, C. Pearce, C. Hillaire-Marcel, D. Calvin Baffin Bay paleoenvironments in the LGM and HS1: Resolving the ice-shelf question. *Mar. Geol.* **402**, 5–16 (2018).

12. R. Hesse, I. Klauck, S. Khodabakhsh, D. Piper, Continental slope sedimentation adjacent to an ice margin. III. The upper Labrador Slope. *Mar. Geol.* **155**, 249–276 (1999).

13. J. N. Bassis, S. V. Petersen, L. Mac Cathles, Heinrich events triggered by ocean forcing and modulated by isostatic adjustment. *Nature* **542**, 332–334 (2017).

14. L. Max, D. Nürnberg, C. M. Chiessi, M. M. Lenz, S. Mulitza, Subsurface ocean warming preceded Heinrich Events. *Nat. Commun.* **13**, 4217 (2022).

15. F. A. Ziemen, M.-L. Kapsch, M. Klockmann, U. Mikolajewicz, Heinrich events show two-stage climate response in transient glacial simulations. *Clim. Past* **15**, 153–168 (2019).

16. D. Roche, D. Paillard, E. Cortijo, Constraints on the duration and freshwater release of Heinrich event 4 through isotope modelling. *Nature* **432**, 379–382 (2004).

17. J. D. Stanford, E. J. Rohling, S. Bacon, A. P. Roberts, F. E. Grousset, M. Bolshaw, A new concept for the paleoceanographic evolution of Heinrich event 1 in the North Atlantic. *Quat. Sci. Rev.* **30**, 1047–1066 (2011).

18. D. A. Hodell, J. A. Nicholl, T. R. R. Bontognali, S. Danino, J. Dorador, J. A. Dowdeswell, J. Einsle, H. Kuhlmann, B. Martrat, M. J. Mleneck-Vautravers, F. J. Rodríguez-Tovar, U. Röhl, Anatomy of Heinrich Layer 1 and its role in the last deglaciation. *Paleoceanography* **32**, 284–303 (2017).

19. T. Chen, L. F. Robinson, M. P. Beasley, L. M. Claxton, M. B. Andersen, L. J. Gregoire, J. Wadham, D. J. Fornari, K. S. Harpp, Ocean mixing and ice-sheet control of seawater $^{234}U/^{238}U$ during the last deglaciation. *Science* **354**, 626–629 (2016).

20. I. K. Seierstad, P. M. Abbott, M. Bigler, T. Blunier, A. J. Bourne, E. Brook, S. L. Buchardt, C. Buizert, H. B. Clausen, E. Cook, D. Dahl-Jensen, S. M. Davies, M. Guillevic, S. J. Johnsen, D. S. Pedersen, T. J. Popp, S. O. Rasmussen, J. P. Severinghaus, A. Svensson, B. M. Vinther, Consistently dated records from the Greenland GRIP, GISP2 and NGRIP ice cores for the past 104 ka reveal regional millennial-scale δ^{18}O gradients with possible Heinrich event imprint. *Quat. Sci. Rev.* **106**, 29–46 (2014).

21. M. Margold, C. R. Stokes, C. D. Clark, Ice streams in the Laurentide Ice Sheet: Identification, characteristics and comparison to modern ice sheets. *Earth Sci. Rev.* **143**, 117–146 (2015).

22. K. A. Refsnider, G. H. Miller, C. Hillaire-Marcel, M. L. Fogel, B. Ghaleb, R. Bowden, Subglacial carbonates constrain basal conditions and oxygen isotopic composition of the Laurentide Ice Sheet over Arctic Canada. *Geology* **40**, 135–138 (2012).

23. K. A. Refsnider, G. H. Miller, M. L. Fogel, B. Fréchette, R. Bowden, J. T. Andrews, G. L. Farmer, Subglacially precipitated carbonates record geochemical interactions and pollen preservation at the base of the Laurentide Ice Sheet on central Baffin Island, eastern Canadian Arctic. *Quatern. Res.* **81**, 94–105 (2014).

24. S. J. Marshall, P. U. Clark, Basal temperature evolution of North American ice sheets and implications for the 100-kyr cycle. *Geophys. Res. Lett.* **29**, 67-1–67-4 (2002).

25. G. K. C. Clarke, U. Nitsan, W. S. B. Paterson, Strain heating and creep instability in glaciers and ice sheets. *Rev. Geophys.* **15**, 235–247 (1977).

26. C. Ritz, Time dependent boundary conditions for calculating of temperature fields in ice sheets, in *The Physical Basis of Ice Sheet Modeling* (International Association of Hydrological Sciences, 1987), pp. 207–216.

27. C. Hillaire-Marcel, G. Bilodeau, Instabilities in the Labrador Sea water mass structure during the last climatic cycle. *Can. J. Earth Sci.* **37**, 795–809 (2000).

28. J. Lynch-Stieglitz, M. W. Schmidt, L. Gene Henry, W. B. Curry, L. C. Skinner, S. Mulitza, R. Zhang, P. Chang, Muted change in Atlantic overturning circulation over some glacial-aged Heinrich events. *Nat. Geosci.* **7**, 144–150 (2014).

29. D. E. Sugden, Glacial erosion by the Laurentide Ice Sheet. *J. Glaciology* **20**, 367–391 (1978).

30. E. Brouard, P. Lajeunesse, Maximum extent and decay of the Laurentide Ice Sheet in Western Baffin Bay during the Last glacial episode. *Sci. Rep.* **7**, 10711 (2017).

31. I. Joughin, S. Tulaczyk, Positive mass balance of the Ross Ice Streams, West Antarctica. *Science* **295**, 476–480 (2002).

32. D. I. Benn, A. C. Fowler, I. Hewitt, H. Sevestre, A general theory of glacier surges. *J. Glaciol.* **65**, 701–716 (2019).

33. M. Margold, C. R. Stokes, C. D. Clark, J. Kleman, Ice streams in the Laurentide Ice Sheet: A new mapping inventory. *J. Maps* **11**, 380–395 (2015).

34. A. S. Dalton, S. A. Finkelstein, S. L. Forman, P. J. Barnett, T. Pico, J. X. Mitrovica, Was the Laurentide Ice Sheet significantly reduced during Marine Isotope Stage 3? *Geology* **47**, 111–114 (2019).

35. E. J. Gowan, X. Zhang, S. Khosravi, A. Rovere, P. Stocchi, A. L. C. Hughes, R. Gyllencreutz, J. Mangerud, J.-I. Svendsen, G. Lohmann, A new global ice sheet reconstruction for the past 80 000 years. *Nat. Commun.* **12**, 1199 (2021).

36. G. H. Denton, R. F. Anderson, J. R. Toggweiler, R. L. Edwards, J. M. Schaefer, A. E. Putnam, The last glacial termination. *Science* **328**, 1652–1656 (2010).

37. A. Vaks, O. S. Gutareva, S. F. M. Breitenbach, E. Avirmed, A. J. Mason, A. L. Thomas, A. V. Osinzev, A. M. Kononov, G. M. Henderson, Speleothems reveal 500,000-year history of Siberian permafrost. *Science* **340**, 183–6 (2013).

38. A. C. Mix, W. F. Ruddiman, Oxygen-isotope analyses and Pleistocene ice volumes. *Quatern. Res.* **21**, 1–20 (1984).

39. J. T. Andrews, J. D. Ives, G. K. Guennel, J. L. Wray, An Early Tertiary outcrop in North-Central Baffin Island, Northwest Territories, Canada: Environment and significance. *Can. J. Earth Sci.* **9**, 233–238 (1972).

40. D. Lacelle, B. Lauriol, I. D. Clark, Origin, age, and paleoenvironmental significance of carbonate precipitates from a granitic environment, Akshayuk Pass, southern Baffin Island, Canada. *Can. J. Earth Sci.* **44**, 61–79 (2007).

41. M. A. Wadleigh, J. Veizer, C. Brooks, Strontium and its isotopes in Canadian rivers: Fluxes and global implications. *Geochim. Cosmochim. Acta* **49**, 1727–1736 (1985).

42. R. H. McNutt, S. K. Frape, P. Fritz, M. G. Jones, I. M. MacDonald, The $^{87}Sr^{86}Sr$ values of Canadian Shield brines and fracture minerals with applications to groundwater mixing, fracture history, and geochronology. *Geochim. Cosmochim. Acta* **54**, 205–215 (1990).

43. D. Bottomley, A review of theories on the origins of saline waters and brines in the Canadian Precambrian Shield. *Tech. Rep. 17*, Atomic Energy Control Board, Ottawa, Canada (1996).

44. P. Méjean, D. L. Pinti, M. Larocque, B. Ghaleb, G. Meyzonnat, S. Gagné, Processes controlling ^{234}U and ^{238}U isotope fractionation and helium in the groundwater of the St. Lawrence Lowlands, Quebec: The potential role of natural rock fracturing. *Appl. Geochem.* **66**, 198–209 (2016).

45. R. M. Dunk, R. A. Mills, W. J. Jenkins, A reevaluation of the oceanic uranium budget for the Holocene. *Chem. Geol.* **190**, 45–67 (2002).

46. M. Gascoyne, High levels of uranium and radium in groundwaters at Canada's Underground Research Laboratory, Lac du Bonnet, Manitoba, Canada. *Appl. Geochem.* **4**, 577–591 (1989).

47. C. J. Batchelor, I. J. Orland, S. A. Marcott, R. Slaughter, R. L. Edwards, P. Zhang, X. Li, H. Cheng, Distinct permafrost conditions across the last two glacial periods in midlatitude North America. *Geophys. Res. Lett.* **46**, 13318–13326 (2019).

48. J. B. Murton, R. Peterson, J.-C. Ozouf, Bedrock fracture by ice segregation in cold regions. *Science* **314**, 1127–1129 (2006).

49. P. Méjean, D. L. Pinti, B. Ghaleb, M. Larocque, Fracturing-induced release of radiogenic ^{4}He and ^{234}U into groundwater during the last deglaciation: An alternative source to crustal helium fluxes in periglacial aquifers. *Water Resour. Res.* **53**, 5677–5689 (2017).

50. T. F. Kraemer, T. P. Brabets, Uranium isotopes ($^{234}U/^{238}U$) in rivers of the Yukon Basin (Alaska and Canada) as an aid in identifying water sources, with implications for monitoring hydrologic change in arctic regions. *Hydrgeol. J.* **20**, 469–481 (2012).

51. S. Frape, P. Fritz, R. McNutt, Water-rock interaction and chemistry of groundwaters from the Canadian Shield. *Geochim. Cosmochim. Acta* **48**, 1617–1627 (1984).

52. S. K. Frape, P. Fritz, The chemistry and isotopic composition of saline groundwaters from the Sudbury Basin, Ontario. *Can. J. Earth Sci.* **19**, 645–661 (1982).

53. G. S. Boulton, T. Slot, K. Blessing, P. Glasbergen, T. Leijnse, K. van Gijssel, Deep circulation of groundwater in overpressured subglacial aquifers and its geological consequences. *Quat. Sci. Rev.* **12**, 739–745 (1993).

54. I. D. Clark, M. Douglas, K. Raven, D. Bottomley, Recharge and preservation of Laurentide glacial melt water in the Canadian shield. *Ground Water* **38**, 735–742 (2000).

55. R. L. Stotler, S. K. Frape, T. Ruskeeniemi, P. Pitkänen, D. W. Blowes, The interglacial-glacial cycle and geochemical evolution of Canadian and Fennoscandian Shield groundwaters. *Geochim. Cosmochim. Acta* **76**, 45–67 (2012).

56. J.-M. Lemieux, E. A. Sudicky, Simulation of groundwater age evolution during the Wisconsinian glaciation over the Canadian landscape. *Environ. Fluid Mech.* **10**, 91–102 (2010).

57. J. M. Lemieux, E. A. Sudicky, W. R. Peltier, L. Tarasov, Dynamics of groundwater recharge and seepage over the Canadian landscape during the Wisconsinian glaciation. *J. Geophys. Res. Earth* **113**, F01011 (2008).

58. S.-E. Lauritzen, J. E. Mylroie, Results of a speleothem U/Th dating reconnaissance from the Helderberg Plateau, New York. *J. Caves Karst Stud.* **62**, 20–26 (2000).

59. D. Lacelle, B. Lauriol, G. Zazula, B. Ghaleb, N. Utting, I. D. Clark, Timing of advance and basal condition of the Laurentide Ice Sheet during the last glacial maximum in the Richardson Mountains, NWT. *Quat. Res.* **80**, 274–283 (2013).

60. N. Biller-Celander, J. D. Shakun, D. McGee, C. I. Wong, A. V. Reyes, B. Hardt, I. Tal, D. C. Ford, B. Lauriol, Increasing Pleistocene permafrost persistence and carbon cycle conundrums inferred from Canadian speleothems. *Sci. Adv.* **7**, eabe5799 (2021).

61. S. A. Ewing, J. B. Paces, J. A. O'Donnell, M. T. Jorgenson, M. Z. Kanevskiy, G. R. Aiken, Y. Shur, J. W. Harden, R. Striegl, Uranium isotopes and dissolved organic carbon in loess permafrost: Modeling the age of ancient ice. *Geochim. Cosmochim. Acta* **152**, 143–165 (2015).

62. T. Blackburn, G. H. Edwards, S. Tulaczyk, M. Scudder, G. Piccione, B. Hallet, N. McLean, J. C. Zachos, B. Cheney, J. T. Babbe, Ice retreat in Wilkes Basin of East Antarctica during a warm interglacial. *Nature* **583**, 554–559 (2020).

63. J. H. Chen, R. L. Edwards, G. J. Wasserburg, ^{238}U, ^{234}U and ^{232}Th in seawater. *Earth Planet. Sci. Lett.* **80**, 241–251 (1986).

64. E. W. Wolff, H. Fischer, R. Röthlisberger, Glacial terminations as southern warmings without northern control. *Nat. Geosci.* **2**, 206–209 (2009).

65. U. Hoff, T. L. Rasmussen, R. Stein, M. M. Ezat, K. Fahl, Sea ice and millennial-scale climate variability in the Nordic seas 90 kyr ago to present. *Nat. Commun.* **7**, 12247 (2016).

66. Q. Simon, C. Hillaire-Marcel, G. St-Onge, J. T. Andrews, North-eastern Laurentide, western Greenland and southern Innuitian ice stream dynamics during the last glacial cycle. *J. Quat. Sci.* **29**, 14–26 (2014).

67. J. T. Andrews, M. E. Kirby, A. Aksu, D. C. Barber, D. Meese, Late Quaternary detrital carbonate (DC-) layers in Baffin Bay marine sediments (67°–74°N): Correlation with Heinrich events in the North Atlantic? *Quat. Sci. Rev.* **17**, 1125–1137 (1998).

68. A. Gilbert, G. E. Flowers, G. H. Miller, K. A. Refsnider, N. E. Young, V. Radić, The projected demise of Barnes Ice Cap: Evidence of an unusually warm 21st century Arctic. *Geophys. Res. Lett.* **44**, 2810–2816 (2017).

69. E. P. Verplanck, G. L. Farmer, J. Andrews, G. Dunhill, C. Millo, Provenance of Quaternary glacial and glacimarine sediments along the southeast Greenland margin. *Earth Planet. Sci. Lett.* **286**, 52–62 (2009).

70. M. H. Walczak, A. C. Mix, E. A. Cowan, S. Fallon, L. K. Fifield, J. R. Alder, J. Du, B. Haley, T. Hobern, J. Padman, S. K. Praetorius, A. Schmittner, J. S. Stoner, S. D. Zellers, Phasing of millennial-scale climate variability in the Pacific and Atlantic Oceans. *Science* **370**, 716–720 (2020).

71. Z. D. Sharp, *Principles of Stable Isotope Geochemistry* (University of New Mexico, ed. 2, 2017).

72. J. Ma, G. Wei, Y. Liu, Z. Ren, Y. Xu, Y. Yang, Precise measurement of stable ($\delta^{88/86}$Sr) and radiogenic (^{87}Sr/^{86}Sr) strontium isotope ratios in geological standard reference materials using MC-ICP-MS. *Chin. Sci. Bull.* **58**, 3111–3118 (2013).

73. C. L. Batchelor, M. Margold, M. Krapp, D. K. Murton, A. S. Dalton, P. L. Gibbard, C. R. Stokes, J. B. Murton, A. Manica, The configuration of Northern Hemisphere ice sheets through the Quaternary. *Nat. Commun.* **10**, 3713 (2019).

74. H. Cheng, R. L. Edwards, C. C. Shen, V. J. Polyak, Y. Asmerom, J. Woodhead, J. Hellstrom, Y. Wang, X. Kong, C. Spötl, X. Wang, E. C. Alexander Jr., Improvements in ^{230}Th dating, ^{230}Th and ^{234}U half-life values, and U-Th isotopic measurements by multi-collector inductively coupled plasma mass spectrometry. *Earth Planet. Sci. Lett.* **371-372**, 82–91 (2013).

75. S. O. Rasmussen, M. Bigler, S. P. Blockley, T. Blunier, S. L. Buchardt, H. B. Clausen, I. Cvijanovic, D. Dahl-Jensen, S. J. Johnsen, H. Fischer, V. Gkinis, M. Guillevic, W. Z. Hoek, J. J. Lowe, J. B. Pedro, T. Popp, I. K. Seierstad, J. P. Steffensen, A. M. Svensson, P. Vallelonga, B. M. Vinther, M. J. C. Walker, J. J. Wheatley, M. Winstrup, A stratigraphic framework for abrupt climatic changes during the Last Glacial period based on three synchronized Greenland ice-core records: Refining and extending the INTIMATE event stratigraphy. *Quat. Sci. Rev.* **106**, 14–28 (2014).

76. P. Vermeesch, On the visualisation of detrital age distributions. *Chem. Geol.* **312–313**, 190–194 (2012).

77. D. L. Phillips, P. L. Koch, Incorporating concentration dependence in stable isotope mixing models. *Oecologia* **130**, 114–125 (2002).

78. P. Vermeesch, IsoplotR: A free and open toolbox for geochronology. *Geosci. Front.* **9**, 1479–1493 (2018).

79. D. Docquier, L. Perichon, F. Pattyn, Representing grounding line dynamics in numerical ice sheet models: Recent advances and outlook. *Surv. Geophys.* **32**, 417–435 (2011).

80. C. F. Brædstrup, D. L. Egholm, S. V. Ugelvig, V. K. Pedersen, Basal shear stress under alpine glaciers: Insights from experiments using the iSOSIA and Elmer/Ice models. *Earth Surf. Dyn.* **4**, 159–174 (2016).

81. S. Tulaczyk, W. B. Kamb, H. F. Engelhardt, Basal mechanics of Ice Stream B, West Antarctica. 2. Undrained-plastic-bed model. *J. Geophys. Res.* **105**, 483–494 (2000).

82. R. L. Hooke, E. C. Alexander Jr., R. J. Gustafson, Temperature profiles in the Barnes Ice Cap, Baffin Island, Canada, and heat flux from the subglacial terrane. *Can. J. Earth Sci.* **17**, 1174–1188 (1980).

83. A. Gilbert, G. E. Flowers, G. H. Miller, B. T. Rabus, W. Van Wychen, A. S. Gardner, L. Copland, Sensitivity of Barnes Ice Cap, Baffin Island, Canada, to climate state and internal dynamics. *J. Geophys. Res. Earth* **121**, 1516–1539 (2016).

84. C. B. Begeman, S. M. Tulaczyk, A. T. Fisher, Spatially variable geothermal heat flux in West Antarctica: Evidence and implications. *Geophys. Res. Lett.* **44**, 9823–9832 (2017).

85. Y.-C. Yen, Review of thermal properties of snow, ice and sea ice, *Tech. Rep. 81–10*, U.S. Army Cold Regions Research and Engineering Laboratory, Hanover, New Hampshire, USA (1981).

86. V. Masson-Delmotte, G. Dreyfus, P. Braconnot, S. Johnsen, J. Jouzel, M. Kageyama, A. Landais, M.-F. Loutre, J. Nouet, F. Parrenin, D. Raynaud, B. Stenni, E. Tuenter, Past temperature reconstructions from deep ice cores: Relevance for future climate change. *Clim. Past* **2**, 145–165 (2006).

87. E. J. Steig, How well can we parameterize past accumulation rates in polar ice sheets? *Ann. Glaciol.* **25**, 418–422 (1997).

88. J. P. Briner, J. C. Gosse, P. R. Bierman, Applications of cosmogenic nuclides to Laurentide Ice Sheet history and dynamics, in *In Situ-Produced Cosmogenic Nuclides and Quantification of Geological Processes* (Geological Society of America, 2006), vol. 415, pp. 29–41.

89. J. L. Bamber, R. L. Layberry, S. P. Gogineni, A new ice thickness and bed data set for the Greenland ice sheet: 1. Measurement, data reduction, and errors. *J. Geophys. Res. Atmos.* **106**, 33773–33780 (2001).

90. B. Hallet, Deposits formed by subglacial precipitation of $CaCO_3$. *GSA Bulletin* **87**, 1003–1015 (1976).

91. G. H. Miller, A. P. Wolfe, E. J. Steig, P. E. Sauer, M. R. Kaplan, J. P. Briner, The Goldilocks dilemma: Big ice, little ice, or "just-right" ice in the Eastern Canadian Arctic. *Quat. Sci. Rev.* **21**, 33–48 (2002).

92. J. F. Nye, The response of glaciers and ice-sheets to seasonal and climatic changes. *Proc. R. Soc. Lond. A* **256**, 559–584 (1960).

93. M. R. Koutnik, E. D. Waddington, Well-posed boundary conditions for limited-domain models of transient ice flow near an ice divide. *J. Glaciol.* **58**, 1008–1020 (2012).

94. B. Cowan, J. Carter, D. Forbes, T. Bell, Postglacial sea-level lowstand on Cumberland Peninsula, Baffin Island, Nunavut. *Can. J. Earth Sci.* (2021).

95. R. N. Hiscott, A. E. Aksu, O. B. Nielsen, Provenance and dispersal patterns, Pliocene-Pleistocene section at site 645, Baffin Bay, in *Proceedings of the Ocean Drilling Program, Scientific Results*, S. K. Stewart, Ed. (Ocean Drilling Program, 1989), vol. 105, pp. 31–52.

96. L. A. Neymark, J. B. Paces, High-precision isotope analysis of uranium and thorium by TIMS. *AGU Fall Meeting Abstracts* V11E-04 (2006).

97. B. Hamelin, E. Bard, A. Zindler, R. G. Fairbanks, $^{234}U/^{238}U$ mass spectrometry of corals: How accurate is the U-Th age of the last interglacial period? *Earth Planet. Sci. Lett.* **106**, 169–180 (1991).

98. P. M. Chutcharavan, A. Dutton, A global compilation of U-series-dated fossil coral sea-level indicators for the Last Interglacial period (Marine Isotope Stage 5e). *Earth Syst. Sci. Data* **13**, 3155–3178 (2021).
